# Risk of Subsequent Coronary Heart Disease in Patients Hospitalized for Immune-Mediated Diseases: A Nationwide Follow-Up Study from Sweden

**DOI:** 10.1371/journal.pone.0033442

**Published:** 2012-03-16

**Authors:** Bengt Zöller, Xinjun Li, Jan Sundquist, Kristina Sundquist

**Affiliations:** 1 Center for Primary Health Care Research, Lund University/Region Skåne, Clinical Research Centre, Skåne University Hospital, Malmö, Sweden; 2 Stanford Prevention Research Center, Stanford University School of Medicine, Stanford, California, United States of America; University of Texas at San Antonio, United States of America

## Abstract

**Background:**

Certain immune-mediated diseases (IMDs), such as rheumatoid arthritis and systemic lupus erythematosus, have been linked to cardiovascular disorders. We examined whether there is an association between 32 different IMDs and risk of subsequent hospitalization for coronary heart disease (CHD) related to coronary atherosclerosis in a nationwide follow up study in Sweden.

**Methods and Findings:**

All individuals in Sweden hospitalized with a main diagnosis of an IMD (n = 336,479) without previous or coexisting CHD, between January 1, 1964 and December 31 2008, were followed for first hospitalization for CHD. The reference population was the total population of Sweden. Standardized incidence ratios (SIRs) for CHD were calculated. Overall risk of CHD during the first year after hospitalization for an IMD was 2.92 (95% CI 2.84–2.99). Twenty-seven of the 32 IMDs studied were associated with an increased risk of CHD during the first year after hospitalization. The overall risk of CHD decreased over time, from 1.75 after 1–5 years (95% CI 1.73–1.78), to 1.43 after 5–10 years (95% CI 1.41–1.46) and 1.28 after 10+ years (95% CI 1.26–1.30). Females generally had higher SIRs than males. The IMDs for which the SIRs of CDH were highest during the first year after hospitalization included chorea minor 6.98 (95% CI 1.32–20.65), systemic lupus erythematosus 4.94 (95% CI 4.15–5.83), rheumatic fever 4.65 (95% CI 3.53–6.01), Hashimoto's thyroiditis 4.30 (95% CI 3.87–4.75), polymyositis/dermatomyositis 3.81 (95% CI 2.62–5.35), polyarteritis nodosa 3.81 (95% CI 2.72–5.19), rheumatoid arthritis 3.72 (95% CI 3.56–3.88), systemic sclerosis 3.44 (95% CI 2.86–4.09), primary biliary cirrhosis 3.32 (95% CI 2.34–4.58), and autoimmune hemolytic anemia 3.17 (95% CI 2.16–4.47).

**Conclusions:**

Most IMDs are associated with increased risk of CHD in the first year after hospital admission. Our findings suggest that many hospitalized IMDs are tightly linked to coronary atherosclerosis.

## Introduction

Coronary heart disease (CHD) and myocardial infarction are major causes of morbidity and mortality worldwide [1]. During recent years it has become clear that systemic inflammation can enhance atherogenesis [2],[3],[4]. Immune-mediated diseases (IMDs) are a heterogeneous group of disorders that are characterized by acute or chronic inflammation. Certain IMDs have been linked to an increased risk for cardiovascular disease (CVD) [5]. IMDs may increase the CVD risk through several mechanisms such as autoantibodies, autoantigens, autoreactive lymphocytes, epigenetic mechanisms, and inflammatory components driving the formation, progression and rupture of atherosclerotic plaques [2],[3],[4],[5],[6],[7]. Inflammation may also modulate the thrombotic responses by upregulating procoagulants, downregulating anticoagulants and suppressing fibrinolysis [7]. It is therefore not surprising that inflammatory IMDs such as rheumatoid arthritis (RA) [3],[5],[6],[8],[9],[10],[11],[12] and systemic lupus erythematosus (SLE) [3],[5],[6],[8],[13],[14],[15] have been linked to an increased risk of CVD. An enhanced atherogenesis has also been suggested in several other IMDs such as Sjögren's disease [3],[5],[6],[16], systemic vasculitis [3],[5], inflammatory bowel disease [3],[5],[8] [17], and psoriasis [8], [18].

We postulated that not only immune-mediated diseases such as RA and SLE, but also a number of other less well studied IMDs or related diseases have an increased risk of CVD. More specifically we aimed at determining whether IMDs increase the risk for hospitalized CHD related to coronary atheroslerosis. In a nationwide follow-up study from 1964–2008 we have estimated the risk of hospitalization with CHD in patients hospitalized with 32 different immune-mediated diseases whithout previous or coexisting CHD.

## Methods

### MigMed 2 Database

The study was approved by the Ethics Committee at Lund University and recommendations of the Declaration of Helsinki were complied with. Informed consent was waived as a requirement by the ethics committee. Data used in this study represented information on all individuals registered as residents of Sweden. It included individual-level information on age, sex, occupation, geographic region of residence, hospital diagnoses, and dates of hospital admissions in Sweden (1964–2008), as well as date of emigration, and date and cause of death. The datasources were several national Swedish data registers (reviewed by Rosen and Hakulinen) [19], including the Swedish National Population and Housing Census (1960–1990), the Total Population Register, the Multi-Generation register, and the Swedish Hospital Discharge register (1964–2008) [20], provided to us by Statistics Sweden and the National Board of Health and Welfare.

Information retrieved from the various registers was linked, at the individual level, via the national 10-digit personal identification number assigned to each resident of Sweden for his or her lifetime. Registration numbers were replaced by serial numbers to preserve anonymity. As well as being used to track all records in the dataset at the individual level, these serial numbers were used to check that individuals with hospital diagnoses of CHD appeared only once in the data set (for the first hospital diagnosis of CHD during the study period).

The follow-up period for analysis of these data in the present study started on January 1, 1964 and continued until hospitalisation for CHD, death, emigration, or the end of the study period (December 31, 2008). Data for first hospitalisation for CHD (main or secondary diagnosis) during the study period were retrieved from the Hospital Discharge Register (1964–2008), provided to us by the National Board of Health and Welfare. This register does not include data for hospital outpatients or patients treated at primary health care centres.

### Predictor variable

The predictor variable was first hospitalization for a main diagnosis of an IMD, diagnosed according to ICD-7, ICD-8, ICD-9 and ICD-10 (Table S1). IMD patients with CHD (main or secondary diagnosis) before or at the same time as first hospitalization for IMD (n = 32,352) were excluded.

### Outcome variable

Diagnosis of CHD was based on the 7th, 8th, 9th and 10th revisions of the International Classification of Diseases (ICD-7, ICD-8, ICD-9 and ICD-10). Cases with a main or secondary diagnosis of CHD were identified using the following ICD codes: 420 (ICD-7); 410-410 (ICD-8); 410-414 (ICD-9); and I20–I25 (ICD-10).

ICD 7

420.0: CHD

420.1: acute cardiac infarction

420.2: angina pectoris

420.9: old cardiac infarction

ICD 8

410: acute cardiac infarction

411: other acute and subacute forms of CHD

412: old cardiac infarction or chronic CHD

413: angina pectoris

414: asymptomatic CHD

ICD 9

410: acute cardiac infarction

411: other acute and subacute forms of CHD

412: old cardiac infarction

413: angina pectoris

414: other forms of chronic CHD

ICD 10

I20: angina pectoris

I21: acute cardiac infarction

I22: reinfarction (within 4 weeks)

I23: complications due to acute cardiac infarction

I24: other acute forms of CHD

I25: chronic CHD

### Individual-level variables adjusted for in the model

The individual-level variables included in the analysis were sex, age, time period, geographic region of residence, socioeconomic status (SES) and comorbidity.

Sex: male or female.

Time period: time was divided into five periods in order to allow for adjustment for any change in incidence over time: 1964–1973, 1974–1983, 1984–1993, 1994–2003, and 2003–2008.

Age was divided into 5-year categories. Subjects of all ages were included in the study. Geographic region of residence was included as an individual-level variable to adjust for possible differences in hospital admissions for CHD between different geographic regions in Sweden. It was categorized as 1) large city (city with a population of >200,000 (i.e., Stockholm, Gothenburg or Malmo), 2) Southern Sweden (both rural and urban), and 3) Northern Sweden (both rural and urban).

Occupation was used as a proxy for SES. Occupational data were retrieved from national census records. We classified each individual's occupation into one of six categories: 1) manual worker, 2) lower-level employee, 3) middle-level employee/professional, 4) self-employed, 5) farmer, and 6) other. Homemakers and students without an occupation were categorized on the basis of their father's or mother's occupation. If that was not possible, they were included in the “other” category. Individuals without paid employment were also included in the “other” category. For individuals aged <20 years, parental occupation was used.

Comorbidity was defined as the first hospitalization with a main or secondary diagnosis at follow up from 1964–2008) of the following: 1) chronic lower respiratory diseases (500, 501 and 502 (ICD-7), 490–493 (ICD-8), 490–496 (ICD-9) and J40–J49 (ICD-10)); 2) obesity (287.00 and 287.99 (ICD-7), 277.99 (ICD-8), 278A (ICD-9) and E65–E68 (ICD-10)); 3) alcoholism (307, 322 and 581 (ICD-7), 291, 303 and 571 (ICD-8), 291 and 303 (ICD-9) and F10 and K70 (ICD-10)); 4) type 2 diabetes mellitus (260 (age>29 yr) (ICD-7), 250 (age>29 yr) (ICD-8), 250 (age>29 yr) (ICD-9) and E11–E14 (ICD-10); 5) hypertension (440–447 (ICD-7), 400 and 402–404 (ICD-8), 401–405 (ICD-9) and I10–I15 (ICD-10)); 6) atrial fibrillation (433.12 and 433.13 (ICD-7), 427.92 (ICD-8), 427D (ICD-9) and I48 (ICD-10)); 7) heart failure (434.10, 434.20 and 782.40 (ICD-7), 427.00, 427.10, 428.99 and 782.40 (ICD-8), 428 (ICD-9) and I50 (ICD-10)); 8) renal disease (590–601 and 757.10 (ICD-7), 580–591 and 753.1 (ICD-8), 580–591 and 753B (ICD-9) and N00–N19, Q61 (ICD-10)).

### Statistical analysis

Person-years of risk (i.e., number of persons at risk multiplied by time at risk) were calculated from the time at which subjects were included in the study (in 1964 or later) until first hospitalization for a main or secondary diagnosis of CHD, death, emigration, or the end of the study period (December 31, 2008). Person-years for IMD patients (without main or secondary diagnosis of CHD before or at the same time as first hospitalization) were counted from discharge of the first hospitalization for IMD. The expected number of cases was based on the number of cases in the reference group. SIRs were calculated as the ratio of observed (O) and expected (E) number of CHD cases using the indirect standardization method [21]:
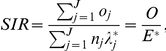
where 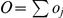 denotes the total observed number of cases in the study group; 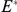 (expected number of cases) is calculated by applying stratum-specific standard incidence rates (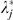) obtained from the reference group to the stratum-specific person-years (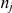) of risk for the study group; 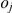 represents the observed number of cases that the cohort subjects contribute to the jth stratum; and J represents the strata defined by cross-classification of the different adjustment variables: age, sex, time period, SES, geographic region of residence, and comorbidity [21]. Ninety-five percent confidence intervals (95% CIs) were calculated assuming a Poisson distribution [21]. All analyses were performed using SAS version 9.2 (SAS Institute, Cary, NC, USA).

## Results

Table S2 shows the number of people in the study who were admitted to hospital with any of the selected IMDs during the study period. Totally 32,352 IMD patients, with a CHD diagnosis before or at the same time as the first hospitalization for IMD, were excluded from the study. After this exclusion, a total of 336,479 patients hospitalized with an IMD (128,536 males and 207,943 females) remained in the study (Table S2). The three most common IMDs were rheumatoid arthritis (62,064 cases), Graves' disease (40,557) and ulcerative colitis (29,698).

Table 1 shows the total number of CHD cases in the entire population (1,934,822 individuals). Of these, 56,135 had had a previous hospitalization for IMDs. The comorbidities (defined as main or second hospital diagnosis) adjusted for are presented in Table 1. The risk of CHD was increased during the first year after hospitalization for 27 of the 32 IMDs studied (Table 2). The overall risk of CHD during the first year after hospitalization for an IMD was 2.92 (95% confidence interval (CI) 2.84–2.99). The overall risk of CHD decreased over time, from 1.75 after 1–5 years (95% CI 1.73–1.78), to 1.43 after 5–10 years (95% CI 1.41–1.46) and 1.28 after 10+ years (95% CI 1.26–1.30).

**Table 1:** Number of cases of coronary heart disease (CHD), 1964–2008.

	All CHD events		Subsequent CHD events of IMD patients	
Characteristics	No.	%	No.	%
Gender				
Men	1109456	57.3	22270	39.7
Women	825366	42.7	33865	60.3
Age at diagnosis (yrs)				
<50	66777	3.5	1670	3.0
50–59	184444	9.5	3963	7.1
60–69	382388	19.8	9207	16.4
70–79	612816	31.7	18306	32.6
> = 80	688397	35.6	22989	41.0
Period of diagnosis (yrs)				
1964–73	355086	18.4	1908	3.4
1974–83	497005	25.7	10221	18.2
1984–93	472520	24.4	17089	30.4
1994–03	424548	21.9	18546	33.0
2004–08	185663	9.6	8371	14.9
Socioeconomic status				
Farmers	186832	9.7	5225	9.3
Self-employed	130747	6.8	3700	6.6
Professionals	79564	4.1	2099	3.7
White collar workers	383918	19.8	13735	24.5
Workers	754431	39.0	25003	44.5
Others	399330	20.6	6373	11.4
Hospitalization for obesity				
Yes	4566	0.2	226	0.4
No	1930256	99.8	55909	99.6
Hospitalization for alcoholism				
Yes	41752	2.2	1509	2.7
No	1893070	97.8	54626	97.3
Hospitalization for chronic lower respiratory diseases				
Yes	82665	4.3	3912	7.0
No	1852157	95.7	52223	93.0
Hospitalization for hypertension				
Yes	43046	2.2	1783	3.2
No	1891776	97.8	54352	96.8
Hospitalization for diabetes type II				
Yes	115547	6.0	4963	8.8
No	1819275	94.0	51172	91.2
Hospitalization for artrial flutter				
Yes	122027	6.3	5171	9.2
No	1812795	93.7	50964	90.8
Hospitalization for heart failure				
Yes	289675	15.0	11796	21.0
No	1645147	85.0	44339	79.0
Hospitalization for renal disease				
Yes	62748	3.2	3606	6.4
No	1872074	96.8	52529	93.6
All	1934822	100.0	56135	100.0

**Table 2:** SIR for subsequent CHD of patients with IMD.

	Follow-up interval (years)																			
	<1				1–5				5–10				> = 10				All			
Immune-mediated diseases	O	SIR	95% CI		O	SIR	95% CI		O	SIR	95% CI		O	SIR	95% CI		O	SIR	95% CI	
Addison's disease	36	**3.06**	**2.14**	**4.24**	90	**1.78**	**1.43**	**2.18**	51	**1.38**	**1.03**	**1.81**	35	1.03	0.71	1.43	212	**1.59**	**1.38**	**1.82**
Amyotrophic lateral sclerosis	153	**2.47**	**2.10**	**2.90**	183	**2.21**	**1.90**	**2.56**	65	**1.43**	**1.11**	**1.83**	81	1.23	0.97	1.52	482	**1.88**	**1.72**	**2.06**
Ankylosing spondylitis	53	**2.61**	**1.95**	**3.41**	215	**1.82**	**1.58**	**2.08**	170	**1.38**	**1.18**	**1.61**	415	**1.12**	**1.02**	**1.24**	853	**1.35**	**1.26**	**1.45**
Autoimmune hemolytic anemia	32	**3.17**	**2.16**	**4.47**	61	**1.50**	**1.15**	**1.93**	43	**1.61**	**1.17**	**2.17**	42	1.31	0.95	1.78	178	**1.63**	**1.40**	**1.88**
Behcet's disease	55	**3.13**	**2.36**	**4.08**	172	**1.82**	**1.56**	**2.11**	146	**1.69**	**1.43**	**1.99**	293	**1.12**	**1.00**	**1.26**	666	**1.45**	**1.34**	**1.56**
Celiac disease	37	**2.57**	**1.81**	**3.54**	116	**1.43**	**1.18**	**1.71**	95	**1.25**	**1.01**	**1.52**	138	0.86	0.72	1.01	386	**1.16**	**1.05**	**1.28**
Chorea minor	3	**6.98**	**1.32**	**20.65**	11	**5.50**	**2.73**	**9.88**	3	2.10	0.40	6.21	4	2.58	0.67	6.67	21	**3.88**	**2.40**	**5.94**
Crohn's disease	135	**2.22**	**1.86**	**2.63**	376	**1.15**	**1.03**	**1.27**	291	0.99	0.88	1.11	502	0.92	0.84	1.01	1304	**1.06**	**1.01**	**1.12**
Diabetes mellitus type I	0				8	**5.56**	**2.37**	**11.00**	16	**4.97**	**2.83**	**8.09**	488	**3.02**	**2.76**	**3.30**	512	**3.08**	**2.82**	**3.36**
Discoid lupus erythematosus	5	1.85	0.58	4.34	36	**2.38**	**1.67**	**3.30**	25	**1.73**	**1.12**	**2.56**	60	**1.68**	**1.29**	**2.17**	126	**1.86**	**1.55**	**2.21**
Grave's disease	474	**2.43**	**2.21**	**2.66**	1737	**1.34**	**1.27**	**1.40**	1621	**1.23**	**1.18**	**1.30**	3391	**1.10**	**1.06**	**1.14**	7223	**1.23**	**1.20**	**1.25**
Hashimoto's thyroiditis	375	**4.30**	**3.87**	**4.75**	903	**2.03**	**1.90**	**2.16**	533	**1.58**	**1.45**	**1.72**	774	**1.39**	**1.30**	**1.49**	2585	**1.81**	**1.74**	**1.88**
Immune thrombocytopenic purpura	46	**2.69**	**1.97**	**3.60**	127	**1.60**	**1.34**	**1.91**	82	**1.40**	**1.11**	**1.73**	86	**1.28**	**1.03**	**1.58**	341	**1.53**	**1.38**	**1.71**
Localized scleroderma	11	1.82	0.90	3.26	50	1.19	0.89	1.57	50	1.19	0.88	1.57	81	1.25	0.99	1.55	192	**1.24**	**1.07**	**1.43**
Lupoid hepatitis	4	2.34	0.61	6.05	8	1.29	0.55	2.55	7	1.54	0.61	3.19	14	0.81	0.44	1.37	33	1.11	0.77	1.56
Multiple sclerosis	150	**2.96**	**2.50**	**3.47**	382	**1.54**	**1.39**	**1.70**	257	**1.30**	**1.14**	**1.47**	330	0.97	0.87	1.08	1119	**1.34**	**1.26**	**1.42**
Myasthenia gravis	47	**2.46**	**1.81**	**3.28**	143	**1.56**	**1.31**	**1.84**	80	1.23	0.97	1.53	93	1.12	0.90	1.37	363	**1.40**	**1.26**	**1.55**
Pernicious anemia	294	**1.90**	**1.69**	**2.13**	1163	**1.25**	**1.18**	**1.32**	1001	**1.33**	**1.25**	**1.42**	1058	**1.43**	**1.35**	**1.52**	3516	**1.36**	**1.32**	**1.41**
Polyarteritis nodosa	40	**3.81**	**2.72**	**5.19**	97	**2.12**	**1.72**	**2.58**	43	1.13	0.82	1.53	68	1.19	0.93	1.51	248	**1.64**	**1.44**	**1.86**
Polymyalgia rheumatica	468	**2.18**	**1.98**	**2.38**	2031	**1.65**	**1.58**	**1.72**	1429	**1.54**	**1.46**	**1.62**	1741	**1.42**	**1.35**	**1.49**	5669	**1.57**	**1.53**	**1.62**
Polymyositis/dermatomyositis	33	**3.81**	**2.62**	**5.35**	76	**2.17**	**1.71**	**2.72**	40	**1.55**	**1.11**	**2.12**	68	**1.55**	**1.20**	**1.96**	217	**1.92**	**1.67**	**2.19**
Primary biliary cirrhosis	37	**3.32**	**2.34**	**4.58**	51	**1.71**	**1.28**	**2.26**	30	1.40	0.95	2.01	43	1.36	0.98	1.83	161	**1.72**	**1.46**	**2.00**
Psoriasis	305	**2.99**	**2.67**	**3.35**	1197	**2.02**	**1.91**	**2.14**	799	**1.52**	**1.42**	**1.63**	1424	**1.35**	**1.28**	**1.42**	3725	**1.64**	**1.59**	**1.69**
Reiter's disease	2	2.02	0.19	7.43	10	1.43	0.68	2.64	9	1.31	0.59	2.50	13	1.69	0.90	2.91	34	**1.51**	**1.04**	**2.11**
Rheumatic fever	58	**4.65**	**3.53**	**6.01**	156	**2.03**	**1.72**	**2.37**	149	**1.62**	**1.37**	**1.90**	434	**1.25**	**1.14**	**1.37**	797	**1.51**	**1.41**	**1.62**
Rheumatoid arthritis	2088	**3.72**	**3.56**	**3.88**	6125	**2.35**	**2.29**	**2.40**	2970	**1.73**	**1.67**	**1.80**	2806	**1.52**	**1.46**	**1.57**	13989	**2.08**	**2.04**	**2.11**
Sarcoidosis	124	**3.11**	**2.59**	**3.71**	322	**1.41**	**1.26**	**1.57**	283	1.09	0.97	1.22	876	1.00	0.94	1.07	1605	**1.15**	**1.09**	**1.20**
Sjögren's syndrome	18	**2.22**	**1.31**	**3.51**	94	**2.04**	**1.65**	**2.50**	45	1.18	0.86	1.59	60	**1.48**	**1.13**	**1.91**	217	**1.63**	**1.42**	**1.87**
Systemic lupus erythematosus	138	**4.94**	**4.15**	**5.83**	357	**2.78**	**2.50**	**3.09**	225	**2.11**	**1.84**	**2.41**	288	**1.60**	**1.42**	**1.79**	1008	**2.27**	**2.14**	**2.42**
Systemic sclerosis	125	**3.44**	**2.86**	**4.09**	317	**1.66**	**1.48**	**1.85**	229	**1.32**	**1.16**	**1.51**	397	**1.20**	**1.08**	**1.32**	1068	**1.46**	**1.37**	**1.55**
Ulcerative colitis	205	**2.07**	**1.80**	**2.38**	756	**1.33**	**1.24**	**1.43**	609	**1.17**	**1.07**	**1.26**	998	**1.08**	**1.01**	**1.15**	2568	**1.21**	**1.17**	**1.26**
Wegener's granulomatosis	333	**2.21**	**1.98**	**2.46**	1417	**1.47**	**1.39**	**1.55**	1220	**1.37**	**1.30**	**1.45**	1747	**1.54**	**1.47**	**1.62**	4717	**1.50**	**1.46**	**1.55**
All	5884	**2.92**	**2.84**	**2.99**	18787	**1.75**	**1.73**	**1.78**	12616	**1.43**	**1.41**	**1.46**	18848	**1.28**	**1.26**	**1.30**	56135	**1.55**	**1.53**	**1.56**

O = observed number of cases; SIR = standardized incidence ratio; CI = confidence interval.

Bold type: 95% CI does not include 1.00.

Adjusted for age, period, socioeconomic status, hospitalization of chronic lower respiratory diseases, obesity, alcohol, hypertension, diabetes, arterial flutter, heart failure, and renal disease.

The risk of CHD was ≥3 during the first year after hospitalization for 13 IMDs (Table 2). For 18 IMDs, the risk of CHD was increased 10+ years after hospitalization, i.e., ankylosing spondylitis, Behçet's disease, type 1 diabetes mellitus, discoid lupus erythematosus, Graves'disease, Hashimoto's thyroiditis, immune thrombocytopenic purpura, pernicious anemia, polymyalgia rheumatica, polymyositis/dermatomyositis, psoriasis, rheumatic fever, rheumatoid arthritis, Sjögren's syndrome, systemic lupus erythematosus, systemic sclerosis, ulcerative colitis and Wegener's granulomatosis (Table 2).

The SIR for CHD was highest among individuals younger than 50 years but was increased also among older IMD patients (Table 3). The overall risk of CHD was increased in both males and females at different times after hospitalization with an IMD (Tables S3 and S4) and in all studied age groups (<50, 50–59, 60–69,70–79 and >80 years) (Tables S5 and S6). The SIR for CHD tended to be slightly higher for females with IMDs than males with IMDs (Tables S3, S4, S5, and S6). The overall risk of CHD for females during the first year after hospitalization for an IMD was 3.06 (95% CI 2.96–3.17) versus 2.72 (95% CI 2.61–2.83) for males.

**Table 3:** SIR for subsequent CHD of patients with IMD after one year of follow-up.

	Age at diagnosis of CHD (years)															
	<50				50–59				60–69				> = 70			
Immune-mediated diseases	O	SIR	95% CI		O	SIR	95% CI		O	SIR	95% CI		O	SIR	95% CI	
Addison's disease	8	1.83	0.78	3.62	19	1.43	0.86	2.23	27	1.33	0.88	1.94	122	**1.46**	**1.21**	**1.74**
Amyotrophic lateral sclerosis	3	1.02	0.19	3.02	29	**2.90**	**1.94**	**4.17**	68	**2.04**	**1.58**	**2.58**	229	**1.55**	**1.36**	**1.76**
Ankylosing spondylitis	59	**1.58**	**1.21**	**2.04**	173	**1.33**	**1.14**	**1.55**	228	**1.19**	**1.04**	**1.36**	340	**1.35**	**1.21**	**1.50**
Autoimmune hemolytic anemia	1	0.63	0.00	3.58	3	0.68	0.13	2.02	18	1.45	0.86	2.29	124	**1.53**	**1.27**	**1.83**
Behcet's disease	48	**1.82**	**1.34**	**2.42**	81	**1.30**	**1.03**	**1.61**	114	**1.29**	**1.06**	**1.55**	368	**1.39**	**1.25**	**1.54**
Celiac disease	12	1.07	0.55	1.88	36	1.19	0.83	1.64	75	1.12	0.88	1.40	226	1.08	0.94	1.23
Chorea minor	1	9.09	0.00	52.11	3	**21.43**	**4.04**	**63.43**	2	3.57	0.34	13.13	12	**2.88**	**1.48**	**5.05**
Crohn's disease	70	0.95	0.74	1.21	173	0.85	0.73	0.99	265	0.93	0.82	1.05	661	**1.09**	**1.01**	**1.18**
Diabetes mellitus type I	453	**3.49**	**3.18**	**3.83**	58	**1.64**	**1.24**	**2.12**	1	0.93	0.00	5.31	0			
Discoid lupus erythematosus	3	1.71	0.32	5.07	16	**2.11**	**1.20**	**3.44**	41	**2.63**	**1.88**	**3.57**	61	**1.52**	**1.16**	**1.95**
Grave's disease	115	**1.27**	**1.05**	**1.52**	425	**1.23**	**1.12**	**1.35**	1084	**1.18**	**1.11**	**1.26**	5125	**1.18**	**1.15**	**1.21**
Hashimoto's thyroiditis	21	1.55	0.96	2.38	133	**2.33**	**1.95**	**2.76**	335	**1.90**	**1.70**	**2.11**	1721	**1.58**	**1.50**	**1.65**
Immune thrombocytopenic purpura	9	1.52	0.69	2.90	19	1.40	0.84	2.20	57	**1.85**	**1.40**	**2.39**	210	**1.36**	**1.18**	**1.55**
Localized scleroderma	1	0.98	0.00	5.62	4	1.12	0.29	2.91	10	0.70	0.33	1.28	166	**1.28**	**1.09**	**1.49**
Lupoid hepatitis	1	0.88	0.00	5.07	6	1.91	0.69	4.19	8	1.39	0.59	2.75	14	0.78	0.43	1.32
Multiple sclerosis	47	**1.94**	**1.43**	**2.59**	151	**1.46**	**1.24**	**1.72**	276	**1.40**	**1.24**	**1.57**	495	1.07	0.98	1.17
Myasthenia gravis	10	**2.19**	**1.04**	**4.04**	17	1.24	0.72	1.99	52	**1.40**	**1.04**	**1.83**	237	**1.28**	**1.13**	**1.46**
Pernicious anemia	3	0.75	0.14	2.21	39	1.37	0.98	1.88	176	1.15	0.99	1.34	3004	**1.34**	**1.29**	**1.39**
Polyarteritis nodosa	9	**3.24**	**1.47**	**6.17**	18	**1.88**	**1.11**	**2.98**	43	**1.59**	**1.15**	**2.14**	138	**1.36**	**1.14**	**1.61**
Polymyalgia rheumatica	78	**2.17**	**1.72**	**2.71**	308	**2.01**	**1.79**	**2.25**	582	**1.45**	**1.34**	**1.58**	4233	**1.51**	**1.47**	**1.56**
Polymyositis/dermatomyositis	4	1.85	0.48	4.79	20	**2.50**	**1.52**	**3.86**	36	**1.62**	**1.13**	**2.24**	124	**1.72**	**1.43**	**2.05**
Primary biliary cirrhosis	5	2.44	0.77	5.74	9	1.15	0.52	2.19	27	1.17	0.77	1.71	83	**1.67**	**1.33**	**2.07**
Psoriasis	121	**1.65**	**1.37**	**1.97**	457	**1.93**	**1.75**	**2.11**	783	**1.65**	**1.54**	**1.77**	2059	**1.49**	**1.42**	**1.55**
Reiter's disease	4	2.21	0.57	5.71	9	1.94	0.88	3.71	12	**2.42**	**1.24**	**4.24**	7	0.69	0.27	1.43
Rheumatic fever	69	**2.04**	**1.59**	**2.58**	139	**1.69**	**1.42**	**2.00**	184	**1.39**	**1.19**	**1.60**	347	**1.30**	**1.16**	**1.44**
Rheumatoid arthritis	110	**2.64**	**2.17**	**3.18**	505	**2.32**	**2.12**	**2.53**	2043	**2.47**	**2.37**	**2.58**	9243	**1.82**	**1.78**	**1.85**
Sarcoidosis	67	1.11	0.86	1.41	187	1.04	0.90	1.20	366	**1.17**	**1.05**	**1.29**	861	**1.07**	**1.00**	**1.14**
Sjögren's syndrome	3	2.33	0.44	6.88	9	1.26	0.57	2.40	51	**2.30**	**1.71**	**3.03**	136	**1.45**	**1.21**	**1.71**
Systemic lupus erythematosus	66	**5.90**	**4.56**	**7.51**	114	**3.04**	**2.51**	**3.65**	200	**2.40**	**2.08**	**2.75**	490	**1.73**	**1.58**	**1.89**
Systemic sclerosis	28	1.35	0.89	1.95	76	**1.41**	**1.11**	**1.76**	187	**1.58**	**1.36**	**1.82**	652	**1.30**	**1.20**	**1.40**
Ulcerative colitis	122	1.11	0.93	1.33	319	1.07	0.95	1.19	474	1.06	0.97	1.16	1448	**1.25**	**1.18**	**1.31**
Wegener's granulomatosis	5	1.38	0.44	3.25	48	**2.08**	**1.54**	**2.77**	279	**1.48**	**1.31**	**1.66**	4052	**1.46**	**1.42**	**1.51**
All	1556	**1.87**	**1.77**	**1.96**	3603	**1.51**	**1.46**	**1.56**	8104	**1.52**	**1.49**	**1.55**	36988	**1.44**	**1.42**	**1.45**

O = observed number of cases; SIR = standardized incidence ratio; CI = confidence interval.

Bold type: 95% CI does not include 1.00.

Adjusted for age, period, socioeconomic status, region of residence, hospitalization of chronic lower respiratory diseases, obesity, alcohol, hypertension, diabetes, arterial flutter, heart failure, and renal disease.

The overall risk of CHD was somewhat lower between 1994 and 2008 (SIR 1.42, 95% CI 1.40–1.44) than between 1964 and 1993 (SIR 1.58, 95% CI 1.56–1.60) (Table S7). This was observed both for females and males (Tables S8 and S9).

Overall risks of CHD were slightly higher for IMD patients who stayed in hospital for less than 7 days (overall SIR 1.63, 95% CI 1.61–1.64), compared to those who stayed 7 days or more (SIR 1.41, 95% CI 1.40–1.44) (Table S10).

## Discussion

The present study is the first nationwide study of immune-mediated diseases and CHD. The results indicate that hospitalized immune-mediated diseases affect the risk of hospitalization for CHD in both males and females in all studied ages. The relative risk of hospitalized CHD during the first year after hospitalization with an IMD was even higher than for many traditional risk factors for CHD [22]. Although the CHD risk declined over time, the overall risk of CHD remained increased for 10 or more years. The results of our study are in line with previous studies linking rheumatoid arthritis [3] [5] [6] [8] [9] [10] [11] [12], systemic lupus erythematosus [3],[5],[6],[8],[13],[14],[15], Sjögren's disease [3],[5],[6],[16], systemic vasculitis [3],[5], inflammatory bowel disease [3] [5] [8] [17], and psoriasis [8],[18] to an increased risk of CVD. However, our study is unique because it includes a comparison of patients with a wide spectrum of IMDs with the general population in a nationwide setting, as well as a long-term follow-up of patients for CHD. Moreover, we also found a number of novel associations between IMDs and CHD. The results of the present study suggest that CHD due to coronary atherosclerosis is a common feature of several hospitalized IMDs. This risk is not limited to the period immediately following hospital admission: in the case of IMD for which there were sufficient numbers of cases for analysis it was sustained over time.

The increased risk of CHD may have different underlying causes in different IMDs although a general link between systemic inflammation and atherothrombosis is well established [2],[3],[4],[5],[6],[7]. It may be a reflection of more extreme cases of IMDs with severe inflammation, since the patients in our study had been admitted to hospital. The risk was also slightly higher among IMD patients who stayed in hospital for less than 7 days (SIR 1.63), compared to those who stayed 7 days or more (SIR 1.41). Although we cannot explain these findings, it is possible that those patients who stayed for more than a week also received some treatment for CHD risk factors, which could have decreased their CHD risk.

The effects of treatment (corticosteroids promote hemostasis) may contribute to the identified associations [23]. The fact that the risk of CHD decreased over time may suggest that the CHD risk is linked to the inflammatory activity of the IMD, which is likely to decrease over time due to treatment. In line with this hypothesis, several studies have suggested that disease activity is linked with atherosclerosis progression [24],[25]. Moreover, the risk of CHD for IMD patients was somewhat lower between 1994 and 2008 (SIR 1.42) than between 1964 and 1993 (SIR 1.58), which may reflect a general decrease in CHD rates over time or progressively more intensive antiinflammatoric treatment regimes in the recent decades. However, as we lack treatment data, we cannot test this hypothesis.

The present study has certain limitations. For example, we had no data on general cardiovascular risk factors such as body mass index (BMI), smoking, and diet, because it would be unrealistic to gather such data for an entire national population. However, we did adjust for socioeconomic status, which is associated with factors such as smoking. Adjustment was also made for eight different comorbidities (COPD, obesity, alcoholism and alcohol-related liver disease, hypertension, type 2 diabetes mellitus, atrial fibrillation, heart failure, and renal disease). We had no access to outpatient data, which means that only the most severe cases of immune-mediated diseases (i.e. those requiring hospitalization) were included in the analyses. Thus, although we adjusted for comorbidites residual confounding may still be present. However, we excluded all IMD cases with previous or coexisting CHD (n = 32,352) in order to minimize the risk for selection bias, which instead may have lead to an underestimation of the actual CHD risk in IMD patients. In fact, the relative risks for CHD among patients with RA or SLE in the present study are within the range previously reported for CVD for these two immune-mediated diseases [3],[5],[6],[8],[9],[10],[11],[12],[13],[14],[15]. It is therefore likely that risk estimates in the present study are fairly valid. Moreover, the present findings reflect the “real world” and the risk for subsequent CHD among hospitalized IMD patients without previous or coexisting CHD. Another problem might be that not all CHD are hospitalized. However, most cases of acute coronary syndrome should have been treated at hospitals in Sweden during the study period [26]. Moreover, incidence rates were calculated for the whole follow-up period, divided into time periods, and adjustments were made for possible changes in hospitalization rates over time due to different admission criteria.

This study also had a number of strengths. For instance, the study population included all patients hospitalized with IMD (without previous or coexisting CHD) and subsequently with CHD in Sweden during the study period, which eliminated recall bias. Because of the personal identification number assigned to each resident in Sweden, it was possible to trace the records for every subject for the whole follow-up period. Data on occupation were 99.2% complete (1980 and 1990 census), which enabled us to adjust our models for socioeconomic status. A further strength of the present study was the use of validated hospital discharge data. The Hospital Discharge Register has high validity [19],[27],[28],[29], especially for cardiovascular disorders such as stroke, and myocardial infarction, for which approximately 95% of diagnoses have been shown to be correct [27],[28],[29]. Although, the positive predictive value (PPV) may differ between diagnoses in the Swedish Hospital Dicharge Register, the PPV is generally around 85–95% [29].

In summary, the risk of hospitalization for CHD was, for most immune-mediated diseases, found to be significantly increased during the first year after hospitalization. The risk for CHD decreased with time, but for many IMDs persisted for more than 10 years. The findings of the present study suggest that many hospitalized IMDs are tightly linked to coronary atherosclerosis. Further studies need to clarify the mechanisms behind our findings.

## Supporting Information

Table S1 ICD codes of IMD and related conditions.
(DOC)

Table S2 Number of hospitalizations with a main diagnosis of IMD, 1964–2008.
(DOC)

Table S3 SIR for subsequent CHD of male patients with IMD.
(DOC)

Table S4 SIR for subsequent CHD of female patients with IMD.
(DOC)

Table S5 SIR for subsequent CHD of male patients with IMD after one year of follow-up.
(DOC)

Table S6 SIR for subsequent CHD of female patients with IMD after one year of follow-up.
(DOC)

Table S7 SIR for subsequent CHD of patients with IMD after one year of follow-up for time periods 1964–1993 and 1994–2008.
(DOC)

Table S8 SIR for subsequent CHD of male patients with IMD after one year of follow-up for time periods 1964–1993 and 1994–2008.
(DOC)

Table S9 SIR for subsequent CHD of female patients with IMD after one year of follow-up for time periods 1964–1993 and 1994–2008.
(DOC)

Table S10 SIR for all subsequent CHD of patients with IMD hospitalization length <7 days or 7 or more days.
(DOC)
