# Supplementary material for: Risk of Subsequent Coronary Heart Disease in Patients Hospitalized for Immune-Mediated Diseases: A Nationwide Follow-Up Study from Sweden
Source: PLoS One. 2012 Mar 16;7(3):e33442. doi: 10.1371/journal.pone.0033442 (PMC3306397; doi:10.1371/journal.pone.0033442)
Supplement: Table S1 — ICD codes of IMD and related conditions. (DOC) [file pone.0033442.s001.doc]

**Table S1. ICD codes of IMD** and related conditions

|  | **ICD code** | | | |
| --- | --- | --- | --- | --- |
| **Immune-mediated disease** | **ICD-7** | **ICD-8** | **ICD-9** | **ICD-10** |
| Addison’s disease | 274.4 | 255.1 | 255E | E27.1, E27.2 |
| Amyotrophic lateral sclerosis | 356.1 | 348 | 335C | G12.2 |
| Ankylosing spondylitis | 722.1 | 712.4 | 720A | M45, M08.1 |
| Autoimmune hemolytic anemia | 292.2 | 283.90, 283.92 | 283A | D59.0 |
| Behçet’s disease | - | 136.0 | 136B | M35.2 |
| Celiac disease | 286.00 | 269.00, 269.98 | 579A | K90.0 |
| Chorea minor | 402 | 392.9 | 392X | I02.9 |
| Crohn’s disease | 572.00, 572.09 | 563.00 | 555 | K50 |
| Diabetes mellitus type I | 260 (age <20 yr) | 250 (age <20 yr) | 250 (age <20 yr) | E10 (age <20 yr) |
| Discoid lupus erythematosus | 705.4 | 695.4 | 695E | L93.0 |
| Graves’ disease | 252 | 242 | 242 | E05 |
| Hashimoto’s thyroiditis | 253 | 245.1, 243, 244 | 245C, 243, 244 | E00-E03, E06.3 |
| Immune thrombocytopenic purpura | 296 | 287.3 | 287D | D69.3 |
| Localized scleroderma | - | 701.0 | 701A | L94.0 |
| Lupoid hepatitis | 583 | 573.9 | 571EJ | K75.4 |
| Multiple sclerosis | 345 | 340 | 340 | G35 |
| Myasthenia gravis | 744.0 | 733.0 | 358A | G70.0 |
| Pernicious anemia | 290.0 | 281.0 | 281A | D51.0 |
| Polyarteritis nodosa | - | 446.0 | 446A | M30.0 |
| Polymyalgia rheumatica | - | 717.9 | 725 | M315, M35.3 |
| Polymyositis/dermatomyositis | 710.00, 710.01, 726.30 | 716.0, 716.1 | 710D, 710E | M33 |
| Primary biliary cirrhosis | - | - | 571G | K74.3 |
| Psoriasis | 706 | 696 | 696 | L40 |
| Reiter’s disease | - | 099.3, 711.1 | 099D, 711B | M02.3 |
| Rheumatic fever | 400-401 | 390-392 (not 392.9) | 390-392 (not 392X) | I00-02 (not I02.9) |
| Rheumatoid arthritis | 722 (not 722.1) | 712.1, 712.3 | 714 (not 714E, 714X) | M05, M06, M08.0, M08.2 |
| Sarcoidosis | 138.00-138.10 | 135 | 135 | D86 |
| Sjögren’s syndrome | - | 734.90 | 710C | M35.0 |
| Systemic lupus erythematosus | 705.4 | 734.1 | 710A | M32 |
| Systemic sclerosis | 710.0 (not 710.00, 710.01) | 710.0, 734.0 | 710B | M34 |
| Ulcerative colitis | 572.20, 572.21 | 563.10, 569.02 | 556 | K51 |
| Wegener’s granulomatosis | 456 | 446.3 | 446E | M31.3 |
