# Supplementary material for: Risk of Subsequent Coronary Heart Disease in Patients Hospitalized for Immune-Mediated Diseases: A Nationwide Follow-Up Study from Sweden
Source: PLoS One. 2012 Mar 16;7(3):e33442. doi: 10.1371/journal.pone.0033442 (PMC3306397; doi:10.1371/journal.pone.0033442)
Supplement: Table S2 — Number of hospitalizations with a main diagnosis of IMD, 1964–2008. (DOC) [file pone.0033442.s002.doc]

| **Table S2. Number of hospitalizations with a main diagnosis of IMD, 1964-2008*** | | | | | | | | |
| --- | --- | --- | --- | --- | --- | --- | --- | --- |
|  | Men | |  | Women | |  | All | |
| IMD | No. | % |  | No. | % |  | No. | % |
| Addison´s disease | 812 | 0.63 |  | 1161 | 0.56 |  | 1973 | 0.59 |
| Amyotrophic lateral sclerosis | 3869 | 3.01 |  | 3265 | 1.57 |  | 7134 | 2.12 |
| Ankylosing spondylitis | 4136 | 3.22 |  | 1652 | 0.79 |  | 5788 | 1.72 |
| Autoimmune hemolytic anemia | 414 | 0.32 |  | 604 | 0.29 |  | 1018 | 0.30 |
| Behcet´s disease | 2086 | 1.62 |  | 1224 | 0.59 |  | 3310 | 0.98 |
| Celiac disease | 4430 | 3.45 |  | 6556 | 3.15 |  | 10986 | 3.26 |
| Chorea minor | 23 | 0.02 |  | 43 | 0.02 |  | 66 | 0.02 |
| Crohn´s disease | 11378 | 8.85 |  | 12764 | 6.14 |  | 24142 | 7.17 |
| Diabetes mellitus type I | 11904 | 9.26 |  | 10093 | 4.85 |  | 21997 | 6.54 |
| Discoid lupus erythematosus | 158 | 0.12 |  | 386 | 0.19 |  | 544 | 0.16 |
| Grave´s disease | 6666 | 5.19 |  | 33891 | 16.30 |  | 40557 | 12.05 |
| Hashimoto´s thyroiditis | 2131 | 1.66 |  | 8333 | 4.01 |  | 10464 | 3.11 |
| Immune thrombocytopenic purpura | 2077 | 1.62 |  | 2314 | 1.11 |  | 4391 | 1.30 |
| Localized scleroderma | 132 | 0.10 |  | 702 | 0.34 |  | 834 | 0.25 |
| Lupoid hepatitis | 166 | 0.13 |  | 329 | 0.16 |  | 495 | 0.15 |
| Multiple sclerosis | 5071 | 3.95 |  | 9272 | 4.46 |  | 14343 | 4.26 |
| Myasthenia gravis | 1171 | 0.91 |  | 1583 | 0.76 |  | 2754 | 0.82 |
| Pernicious anemia | 4568 | 3.55 |  | 5901 | 2.84 |  | 10469 | 3.11 |
| Polyarteritis nodosa | 662 | 0.52 |  | 575 | 0.28 |  | 1237 | 0.37 |
| Polymyalgia rheumatica | 7163 | 5.57 |  | 14188 | 6.82 |  | 21351 | 6.35 |
| Polymyositis/dermatomyositis | 586 | 0.46 |  | 945 | 0.45 |  | 1531 | 0.46 |
| Primary biliary cirrhosis | 673 | 0.52 |  | 1179 | 0.57 |  | 1852 | 0.55 |
| Psoriasis | 8560 | 6.66 |  | 8294 | 3.99 |  | 16854 | 5.01 |
| Reiter´s disease | 269 | 0.21 |  | 53 | 0.03 |  | 322 | 0.10 |
| Rheumatic fever | 2529 | 1.97 |  | 989 | 0.48 |  | 3518 | 1.05 |
| Rheumatoid arthritis | 17221 | 13.40 |  | 44843 | 21.57 |  | 62064 | 18.45 |
| Sarcoidosis | 5390 | 4.19 |  | 5365 | 2.58 |  | 10755 | 3.20 |
| Sjögren´s syndrome | 136 | 0.11 |  | 1284 | 0.62 |  | 1420 | 0.42 |
| Systemic lupus erythematosus | 1142 | 0.89 |  | 5000 | 2.40 |  | 6142 | 1.83 |
| Systemic sclerosis | 2742 | 2.13 |  | 3058 | 1.47 |  | 5800 | 1.72 |
| Ulcerative colitis | 16113 | 12.54 |  | 13585 | 6.53 |  | 29698 | 8.83 |
| Wegener´s granulomatosis | 4158 | 3.23 |  | 8512 | 4.09 |  | 12670 | 3.77 |
| All | 128536 | 100.00 |  | 207943 | 100.00 |  | 336479 | 100.00 |

*Totally 32,352 IMD patients with CHD before or at the same time as first hospitalization for IMD were excluded.
