# Supplementary material for: Risk of Subsequent Coronary Heart Disease in Patients Hospitalized for Immune-Mediated Diseases: A Nationwide Follow-Up Study from Sweden
Source: PLoS One. 2012 Mar 16;7(3):e33442. doi: 10.1371/journal.pone.0033442 (PMC3306397; doi:10.1371/journal.pone.0033442)
Supplement: Table S3 — SIR for subsequent CHD of male patients with IMD. (DOC) [file pone.0033442.s003.doc]

| **Table S3. SIR for subsequent CHD of male patients with IMD** | | | | | | | | | | | | | | | | | | | | | | | | |
| --- | --- | --- | --- | --- | --- | --- | --- | --- | --- | --- | --- | --- | --- | --- | --- | --- | --- | --- | --- | --- | --- | --- | --- | --- |
|  | Follow-up interval (years) | | | | | | | | | | | | | | | | | | |  |  |  |  |  |
|  | <1 | | | |  | 1-5 | | | |  | 5-10 | | | |  | >=10 | | | |  | All | | | |
| Immune-mediated diseases | O | SIR | 95% CI | |  | O | SIR | 95% CI | |  | O | SIR | 95% CI | |  | O | SIR | 95% CI | |  | O | SIR | 95% CI | |
| Addison´s disease | 20 | **3.72** | **2.27** | **5.75** |  | 40 | **1.83** | **1.30** | **2.49** |  | 24 | 1.49 | 0.95 | 2.21 |  | 15 | 1.01 | 0.57 | 1.67 |  | 99 | **1.70** | **1.38** | **2.07** |
| Amyotrophic lateral sclerosis | 95 | **2.38** | **1.93** | **2.91** |  | 122 | **2.10** | **1.74** | **2.51** |  | 45 | **1.37** | **1.00** | **1.84** |  | 58 | 1.18 | 0.89 | 1.52 |  | 320 | **1.78** | **1.59** | **1.98** |
| Ankylosing spondylitis | 42 | **2.55** | **1.83** | **3.45** |  | 172 | **1.78** | **1.53** | **2.07** |  | 143 | **1.43** | **1.21** | **1.68** |  | 351 | **1.17** | **1.05** | **1.30** |  | 708 | **1.38** | **1.28** | **1.49** |
| Autoimmune hemolytic anemia | 7 | 1.45 | 0.58 | 3.01 |  | 27 | 1.42 | 0.93 | 2.06 |  | 18 | 1.43 | 0.84 | 2.26 |  | 16 | 1.24 | 0.71 | 2.02 |  | 68 | **1.38** | **1.07** | **1.75** |
| Behcet´s disease | 30 | **2.97** | **2.00** | **4.24** |  | 97 | **1.74** | **1.41** | **2.13** |  | 90 | **1.70** | **1.37** | **2.09** |  | 196 | 1.04 | 0.90 | 1.19 |  | 413 | **1.34** | **1.22** | **1.48** |
| Celiac disease | 21 | **2.71** | **1.67** | **4.14** |  | 59 | **1.47** | **1.12** | **1.90** |  | 34 | 0.90 | 0.62 | 1.26 |  | 64 | 0.78 | 0.60 | 1.00 |  | 178 | 1.06 | 0.91 | 1.23 |
| Chorea minor | 1 | **5.88** | **0.00** | **33.72** |  | 4 | **4.26** | **1.11** | **11.00** |  | 1 | 1.43 | 0.00 | 8.19 |  | 1 | 12.50 | 0.01 | 71.65 |  | 7 | **3.70** | **1.47** | **7.67** |
| Crohn´s disease | 65 | **1.85** | **1.43** | **2.36** |  | 197 | 1.03 | 0.89 | 1.19 |  | 156 | 0.91 | 0.77 | 1.07 |  | 274 | 0.85 | 0.75 | 0.95 |  | 692 | 0.96 | 0.89 | 1.03 |
| Diabetes mellitus type I | 0 |  |  |  |  | 2 | 2.47 | 0.23 | 9.08 |  | 6 | **3.92** | **1.41** | **8.59** |  | 244 | **2.76** | **2.43** | **3.13** |  | 252 | **2.78** | **2.45** | **3.14** |
| Discoid lupus erythematosus | 1 | 0.88 | 0.00 | 5.07 |  | 17 | **2.31** | **1.34** | **3.70** |  | 11 | 1.47 | 0.73 | 2.65 |  | 25 | **1.69** | **1.09** | **2.50** |  | 54 | **1.76** | **1.32** | **2.29** |
| Grave´s disease | 96 | **2.14** | **1.74** | **2.62** |  | 336 | 1.10 | 0.98 | 1.22 |  | 371 | **1.19** | **1.07** | **1.31** |  | 688 | 1.02 | 0.95 | 1.10 |  | 1491 | **1.11** | **1.06** | **1.17** |
| Hashimoto´s thyroiditis | 86 | **3.80** | **3.04** | **4.69** |  | 201 | **1.94** | **1.68** | **2.22** |  | 119 | **1.62** | **1.34** | **1.94** |  | 160 | **1.43** | **1.22** | **1.67** |  | 566 | **1.82** | **1.67** | **1.97** |
| Immune thrombocytopenic purpura | 22 | **2.38** | **1.49** | **3.61** |  | 65 | **1.61** | **1.25** | **2.06** |  | 44 | **1.64** | **1.19** | **2.20** |  | 37 | 1.31 | 0.92 | 1.81 |  | 168 | **1.61** | **1.37** | **1.87** |
| Localized scleroderma | 1 | 1.03 | 0.00 | 5.91 |  | 8 | 1.27 | 0.54 | 2.52 |  | 4 | 0.75 | 0.20 | 1.94 |  | 8 | 0.87 | 0.37 | 1.73 |  | 21 | 0.97 | 0.60 | 1.48 |
| Lupoid hepatitis | 4 | **7.55** | **1.96** | **19.52** |  | 1 | 0.51 | 0.00 | 2.94 |  | 3 | 1.52 | 0.29 | 4.49 |  | 6 | 0.65 | 0.23 | 1.42 |  | 14 | 1.02 | 0.56 | 1.71 |
| Multiple sclerosis | 83 | **3.00** | **2.39** | **3.71** |  | 189 | **1.47** | **1.26** | **1.69** |  | 123 | **1.33** | **1.11** | **1.59** |  | 162 | 1.02 | 0.87 | 1.19 |  | 557 | **1.37** | **1.26** | **1.49** |
| Myasthenia gravis | 27 | **2.39** | **1.57** | **3.48** |  | 81 | **1.50** | **1.19** | **1.86** |  | 46 | 1.16 | 0.85 | 1.55 |  | 51 | 1.19 | 0.89 | 1.57 |  | 205 | **1.39** | **1.20** | **1.59** |
| Pernicious anemia | 146 | **1.86** | **1.57** | **2.19** |  | 533 | **1.17** | **1.07** | **1.27** |  | 423 | **1.15** | **1.04** | **1.26** |  | 480 | **1.31** | **1.20** | **1.43** |  | 1582 | **1.25** | **1.19** | **1.31** |
| Polyarteritis nodosa | 20 | **3.16** | **1.93** | **4.90** |  | 50 | **1.88** | **1.39** | **2.48** |  | 28 | 1.14 | 0.76 | 1.65 |  | 42 | 1.14 | 0.82 | 1.54 |  | 140 | **1.48** | **1.25** | **1.75** |
| Polymyalgia rheumatica | 172 | **2.03** | **1.74** | **2.36** |  | 771 | **1.63** | **1.52** | **1.75** |  | 479 | **1.33** | **1.21** | **1.46** |  | 696 | **1.33** | **1.23** | **1.43** |  | 2118 | **1.47** | **1.41** | **1.53** |
| Polymyositis/dermatomyositis | 16 | **3.53** | **2.01** | **5.75** |  | 38 | **2.07** | **1.46** | **2.84** |  | 18 | **1.37** | **0.81** | **2.17** |  | 31 | **1.47** | **1.00** | **2.09** |  | 103 | **1.81** | **1.47** | **2.19** |
| Primary biliary cirrhosis | 20 | **3.84** | **2.34** | **5.94** |  | 22 | **1.95** | **1.22** | **2.95** |  | 7 | 0.96 | 0.38 | 2.00 |  | 15 | 0.94 | 0.53 | 1.56 |  | 64 | **1.61** | **1.24** | **2.06** |
| Psoriasis | 179 | **3.03** | **2.61** | **3.51** |  | 695 | **2.07** | **1.92** | **2.23** |  | 419 | **1.38** | **1.25** | **1.52** |  | 855 | **1.35** | **1.26** | **1.45** |  | 2148 | **1.62** | **1.55** | **1.69** |
| Reiter´s disease | 2 | 2.67 | 0.25 | 9.81 |  | 10 | 1.89 | 0.90 | 3.49 |  | 7 | 1.24 | 0.49 | 2.56 |  | 13 | 1.90 | 1.00 | 3.25 |  | 32 | **1.72** | **1.18** | **2.44** |
| Rheumatic fever | 37 | **4.36** | **3.07** | **6.02** |  | 99 | **1.80** | **1.46** | **2.19** |  | 108 | **1.55** | **1.27** | **1.88** |  | 332 | **1.25** | **1.12** | **1.39** |  | 576 | **1.44** | **1.33** | **1.57** |
| Rheumatoid arthritis | 725 | **3.59** | **3.34** | **3.87** |  | 2099 | **2.29** | **2.19** | **2.39** |  | 995 | **1.64** | **1.54** | **1.74** |  | 1014 | **1.52** | **1.43** | **1.62** |  | 4833 | **2.02** | **1.96** | **2.08** |
| Sarcoidosis | 61 | **3.03** | **2.31** | **3.89** |  | 167 | **1.49** | **1.27** | **1.73** |  | 137 | 1.09 | 0.92 | 1.29 |  | 409 | 0.95 | 0.86 | 1.04 |  | 774 | **1.12** | **1.05** | **1.21** |
| Sjögren´s syndrome | 2 | 1.79 | 0.17 | 6.57 |  | 10 | 1.82 | 0.87 | 3.37 |  | 9 | 2.02 | 0.91 | 3.85 |  | 8 | 1.66 | 0.71 | 3.29 |  | 29 | **1.83** | **1.22** | **2.63** |
| Systemic lupus erythematosus | 37 | **4.13** | **2.91** | **5.70** |  | 92 | **2.29** | **1.85** | **2.81** |  | 66 | **1.93** | **1.49** | **2.45** |  | 77 | **1.63** | **1.29** | **2.04** |  | 272 | **2.08** | **1.84** | **2.35** |
| Systemic sclerosis | 57 | **3.11** | **2.36** | **4.04** |  | 147 | **1.42** | **1.20** | **1.67** |  | 134 | **1.31** | **1.10** | **1.56** |  | 242 | **1.14** | **1.00** | **1.30** |  | 580 | **1.33** | **1.22** | **1.44** |
| Ulcerative colitis | 134 | **2.18** | **1.83** | **2.58** |  | 479 | **1.34** | **1.22** | **1.47** |  | 393 | **1.17** | **1.05** | **1.29** |  | 645 | **1.08** | **1.00** | **1.17** |  | 1651 | **1.22** | **1.16** | **1.28** |
| Wegener´s granulomatosis | 127 | **2.08** | **1.73** | **2.47** |  | 554 | **1.51** | **1.39** | **1.64** |  | 402 | **1.29** | **1.17** | **1.43** |  | 472 | **1.33** | **1.21** | **1.45** |  | 1555 | **1.42** | **1.35** | **1.49** |
| All | 2336 | **2.72** | **2.61** | **2.83** |  | 7384 | **1.67** | **1.63** | **1.71** |  | 4863 | **1.33** | **1.29** | **1.37** |  | 7687 | **1.22** | **1.19** | **1.25** |  | 22270 | **1.46** | **1.44** | **1.48** |
| O = observed number of cases; SIR = standardized incidence ratio; CI = confidence interval. | | | | | | | | | |  |  |  |  |  |  |  |  |  |  |  |  |  |  |  |
| Bold type: 95% CI does not include 1.00. | | | | | | | | | | | | | | | | |  |  |  |  |  |  |  |  |
| Adjusted for age, period, socioeconomic status, hospitalization of chronic lower respiratory diseases, obesity, alcoholism, hypertension, diabetes, arterial flutter, heart failure, and renal disease. | | | | | | | | | | | | | | | | | | | | | | | | |
