# Supplementary material for: Risk of Subsequent Coronary Heart Disease in Patients Hospitalized for Immune-Mediated Diseases: A Nationwide Follow-Up Study from Sweden
Source: PLoS One. 2012 Mar 16;7(3):e33442. doi: 10.1371/journal.pone.0033442 (PMC3306397; doi:10.1371/journal.pone.0033442)
Supplement: Table S4 — SIR for subsequent CHD of female patients with IMD. (DOC) [file pone.0033442.s004.doc]

| **Table S4. SIR for subsequent CHD of female patients with IMD** | | | | | | | | | | | | | | | | | | | | | | | | |  |
| --- | --- | --- | --- | --- | --- | --- | --- | --- | --- | --- | --- | --- | --- | --- | --- | --- | --- | --- | --- | --- | --- | --- | --- | --- | --- |
|  | Follow-up interval (years) | | | | | | | | | | | | | | | | | | |  |  |  |  |  |  |
|  | <1 | | | |  | 1-5 | | | |  | 5-10 | | | |  | >=10 | | | |  | All | | | |  |
| Immune-mediated diseases | O | SIR | 95% CI | |  | O | SIR | 95% CI | |  | O | SIR | 95% CI | |  | O | SIR | 95% CI | |  | O | SIR | 95% CI | |  |
| Addison´s disease | 16 | **2.51** | **1.43** | **4.08** |  | 50 | **1.74** | **1.29** | **2.29** |  | 27 | 1.30 | 0.85 | 1.89 |  | 20 | 1.04 | 0.63 | 1.61 |  | 113 | **1.50** | **1.24** | **1.81** |  |
| Amyotrophic lateral sclerosis | 58 | **2.64** | **2.00** | **3.41** |  | 61 | **2.49** | **1.90** | **3.19** |  | 20 | 1.59 | 0.97 | 2.46 |  | 23 | 1.37 | 0.87 | 2.06 |  | 162 | **2.14** | **1.82** | **2.49** |  |
| Ankylosing spondylitis | 11 | **2.86** | **1.42** | **5.14** |  | 43 | **1.97** | **1.42** | **2.65** |  | 27 | 1.18 | 0.78 | 1.72 |  | 64 | 0.93 | 0.72 | 1.19 |  | 145 | **1.24** | **1.04** | **1.45** |  |
| Autoimmune hemolytic anemia | 25 | **4.73** | **3.05** | **6.99** |  | 34 | **1.57** | **1.09** | **2.20** |  | 25 | **1.78** | **1.15** | **2.63** |  | 26 | 1.36 | 0.89 | 2.00 |  | 110 | **1.83** | **1.50** | **2.21** |  |
| Behcet´s disease | 25 | **3.36** | **2.17** | **4.96** |  | 75 | **1.93** | **1.52** | **2.42** |  | 56 | **1.67** | **1.26** | **2.17** |  | 97 | **1.35** | **1.10** | **1.65** |  | 253 | **1.67** | **1.47** | **1.89** |  |
| Celiac disease | 16 | **2.41** | **1.37** | **3.92** |  | 57 | **1.39** | **1.05** | **1.80** |  | 61 | **1.59** | **1.22** | **2.04** |  | 74 | 0.94 | 0.74 | 1.18 |  | 208 | **1.26** | **1.10** | **1.45** |  |
| Chorea minor | 2 | 7.69 | 0.73 | 28.29 |  | 7 | **6.60** | **2.62** | **13.68** |  | 2 | 2.74 | 0.26 | 10.08 |  | 3 | 2.04 | 0.38 | 6.04 |  | 14 | **3.98** | **2.17** | **6.69** |  |
| Crohn´s disease | 70 | **2.72** | **2.12** | **3.44** |  | 179 | **1.31** | **1.12** | **1.51** |  | 135 | 1.09 | 0.91 | 1.29 |  | 228 | 1.04 | 0.91 | 1.18 |  | 612 | **1.21** | **1.11** | **1.31** |  |
| Diabetes mellitus type I | 0 |  |  |  |  | 6 | **9.52** | **3.43** | **20.87** |  | 10 | **5.92** | **2.82** | **10.92** |  | 244 | **3.33** | **2.92** | **3.77** |  | 260 | **3.44** | **3.03** | **3.88** |  |
| Discoid lupus erythematosus | 4 | 2.53 | 0.66 | 6.55 |  | 19 | **2.45** | **1.48** | **3.84** |  | 14 | **2.01** | **1.10** | **3.38** |  | 35 | **1.68** | **1.17** | **2.34** |  | 72 | **1.94** | **1.52** | **2.44** |  |
| Grave´s disease | 378 | **2.51** | **2.27** | **2.78** |  | 1401 | **1.41** | **1.34** | **1.48** |  | 1250 | **1.25** | **1.18** | **1.32** |  | 2703 | **1.12** | **1.08** | **1.16** |  | 5732 | **1.26** | **1.23** | **1.29** |  |
| Hashimoto´s thyroiditis | 289 | **4.47** | **3.97** | **5.02** |  | 702 | **2.05** | **1.90** | **2.21** |  | 414 | **1.57** | **1.42** | **1.73** |  | 614 | **1.38** | **1.28** | **1.50** |  | 2019 | **1.81** | **1.73** | **1.89** |  |
| Immune thrombocytopenic purpura | 24 | **3.06** | **1.96** | **4.56** |  | 62 | **1.59** | **1.22** | **2.04** |  | 38 | 1.19 | 0.84 | 1.63 |  | 49 | 1.26 | 0.93 | 1.67 |  | 173 | **1.47** | **1.26** | **1.71** |  |
| Localized scleroderma | 10 | 1.97 | 0.94 | 3.63 |  | 42 | 1.18 | 0.85 | 1.59 |  | 46 | 1.25 | 0.92 | 1.67 |  | 73 | 1.31 | 1.03 | 1.65 |  | 171 | **1.28** | **1.10** | **1.49** |  |
| Lupoid hepatitis | 0 |  |  |  |  | 7 | 1.64 | 0.65 | 3.40 |  | 4 | 1.56 | 0.41 | 4.04 |  | 8 | 1.01 | 0.43 | 2.00 |  | 19 | 1.19 | 0.72 | 1.87 |  |
| Multiple sclerosis | 67 | **2.91** | **2.25** | **3.70** |  | 193 | **1.62** | **1.40** | **1.87** |  | 134 | **1.27** | **1.06** | **1.50** |  | 168 | 0.92 | 0.79 | 1.07 |  | 562 | **1.31** | **1.20** | **1.42** |  |
| Myasthenia gravis | 20 | **2.57** | **1.57** | **3.98** |  | 62 | **1.65** | **1.26** | **2.11** |  | 34 | 1.34 | 0.92 | 1.87 |  | 42 | 1.04 | 0.75 | 1.41 |  | 158 | **1.42** | **1.21** | **1.66** |  |
| Pernicious anemia | 148 | **1.94** | **1.64** | **2.28** |  | 630 | **1.32** | **1.22** | **1.43** |  | 578 | **1.51** | **1.39** | **1.64** |  | 578 | **1.55** | **1.43** | **1.68** |  | 1934 | **1.48** | **1.41** | **1.55** |  |
| Polyarteritis nodosa | 20 | **4.77** | **2.91** | **7.38** |  | 47 | **2.45** | **1.80** | **3.26** |  | 15 | 1.12 | 0.62 | 1.85 |  | 26 | 1.29 | 0.84 | 1.89 |  | 108 | **1.90** | **1.56** | **2.29** |  |
| Polymyalgia rheumatica | 296 | **2.27** | **2.02** | **2.54** |  | 1260 | **1.66** | **1.57** | **1.75** |  | 950 | **1.67** | **1.57** | **1.78** |  | 1045 | **1.49** | **1.40** | **1.58** |  | 3551 | **1.64** | **1.59** | **1.70** |  |
| Polymyositis/dermatomyositis | 17 | **4.11** | **2.39** | **6.59** |  | 38 | **2.29** | **1.62** | **3.14** |  | 22 | **1.74** | **1.09** | **2.64** |  | 37 | **1.62** | **1.14** | **2.23** |  | 114 | **2.03** | **1.67** | **2.44** |  |
| Primary biliary cirrhosis | 17 | **2.87** | **1.67** | **4.60** |  | 29 | **1.57** | **1.05** | **2.26** |  | 23 | **1.63** | **1.03** | **2.45** |  | 28 | 1.78 | 1.18 | 2.58 |  | 97 | **1.79** | **1.45** | **2.18** |  |
| Psoriasis | 126 | **2.94** | **2.45** | **3.50** |  | 502 | **1.96** | **1.79** | **2.14** |  | 380 | **1.71** | **1.54** | **1.89** |  | 569 | **1.35** | **1.24** | **1.47** |  | 1577 | **1.67** | **1.59** | **1.76** |  |
| Reiter´s disease | 0 |  |  |  |  | 0 |  |  |  |  | 2 | 1.65 | 0.16 | 6.08 |  | 0 |  |  |  |  | 2 | 0.50 | 0.05 | 1.85 |  |
| Rheumatic fever | 21 | **5.25** | **3.24** | **8.04** |  | 57 | **2.59** | **1.96** | **3.36** |  | 41 | **1.82** | **1.31** | **2.48** |  | 102 | **1.26** | **1.03** | **1.53** |  | 221 | **1.71** | **1.49** | **1.95** |  |
| Rheumatoid arthritis | 1363 | **3.78** | **3.59** | **3.99** |  | 4026 | **2.38** | **2.30** | **2.45** |  | 1975 | **1.79** | **1.71** | **1.87** |  | 1792 | **1.51** | **1.45** | **1.59** |  | 9156 | **2.11** | **2.07** | **2.15** |  |
| Sarcoidosis | 63 | **3.21** | **2.46** | **4.10** |  | 155 | **1.32** | **1.12** | **1.55** |  | 146 | 1.09 | 0.92 | 1.28 |  | 467 | 1.06 | 0.96 | 1.16 |  | 831 | **1.17** | **1.09** | **1.25** |  |
| Sjögren´s syndrome | 16 | **2.29** | **1.30** | **3.73** |  | 84 | **2.07** | **1.65** | **2.56** |  | 36 | 1.07 | 0.75 | 1.49 |  | 52 | **1.46** | **1.09** | **1.91** |  | 188 | **1.61** | **1.39** | **1.86** |  |
| Systemic lupus erythematosus | 101 | **5.32** | **4.33** | **6.46** |  | 265 | **3.00** | **2.65** | **3.39** |  | 159 | **2.20** | **1.87** | **2.57** |  | 211 | **1.58** | **1.38** | **1.81** |  | 736 | **2.35** | **2.19** | **2.53** |  |
| Systemic sclerosis | 68 | **3.76** | **2.92** | **4.77** |  | 170 | **1.95** | **1.67** | **2.27** |  | 95 | **1.34** | **1.08** | **1.64** |  | 155 | **1.29** | **1.10** | **1.52** |  | 488 | **1.65** | **1.51** | **1.80** |  |
| Ulcerative colitis | 71 | **1.90** | **1.48** | **2.40** |  | 277 | **1.31** | **1.16** | **1.47** |  | 216 | **1.16** | **1.01** | **1.33** |  | 353 | **1.07** | **0.96** | **1.19** |  | 917 | **1.20** | **1.12** | **1.28** |  |
| Wegener´s granulomatosis | 206 | **2.30** | **1.99** | **2.63** |  | 863 | **1.44** | **1.35** | **1.54** |  | 818 | **1.41** | **1.32** | **1.51** |  | 1275 | **1.64** | **1.55** | **1.73** |  | 3162 | **1.55** | **1.49** | **1.60** |  |
| All | 3548 | **3.06** | **2.96** | **3.17** |  | 11403 | **1.81** | **1.78** | **1.84** |  | 7753 | **1.50** | **1.47** | **1.54** |  | 11161 | **1.32** | **1.30** | **1.35** |  | 33865 | **1.61** | **1.59** | **1.63** |  |
| O = observed number of cases; SIR = standardized incidence ratio; CI = confidence interval. | | | | | | | | | |  |  |  |  |  |  |  |  |  |  |  |  |  |  |  |  |
| Bold type: 95% CI does not include 1.00. | | | | | | | | | | | | |  |  |  |  |  |  |  |  |  |  |  |  |  |
| Adjusted for age, period, socioeconomic status, hospitalization of chronic lower respiratory diseases, obesity, alcoholism, hypertension, diabetes, arterial flutter, heart failure, and renal disease. | | | | | | | | | | | | | | | | | | | | | | | | |  |
