# Supplementary material for: Risk of Subsequent Coronary Heart Disease in Patients Hospitalized for Immune-Mediated Diseases: A Nationwide Follow-Up Study from Sweden
Source: PLoS One. 2012 Mar 16;7(3):e33442. doi: 10.1371/journal.pone.0033442 (PMC3306397; doi:10.1371/journal.pone.0033442)
Supplement: Table S5 — SIR for subsequent CHD of male patients with IMD after one year of follow-up. (DOC) [file pone.0033442.s005.doc]

| **Table S5. SIR for subsequent CHD of male patients with IMD after one year of follow-up** | | | | | | | | | | | | | | | | | | | |  |
| --- | --- | --- | --- | --- | --- | --- | --- | --- | --- | --- | --- | --- | --- | --- | --- | --- | --- | --- | --- | --- |
|  | Age at diagnosis of CHD (years) | | | | | | | | | | | | | | | | | | |  |
|  | <50 | | | |  | 50-59 | | | |  | 60-69 | | | |  | >=70 | | | |  |
| Immune-mediated diseases | O | SIR | 95% CI | |  | O | SIR | 95% CI | |  | O | SIR | 95% CI | |  | O | SIR | 95% CI | |  |
| Addison´s disease | 5 | 1.94 | 0.61 | 4.56 |  | 12 | 1.42 | 0.73 | 2.49 |  | 10 | 0.99 | 0.47 | 1.83 |  | 52 | **1.64** | **1.22** | **2.15** |  |
| Amyotrophic lateral sclerosis | 3 | 1.15 | 0.22 | 3.40 |  | 22 | **2.63** | **1.64** | **3.98** |  | 59 | **2.15** | **1.64** | **2.78** |  | 141 | **1.38** | **1.17** | **1.63** |  |
| Ankylosing spondylitis | 52 | **1.57** | **1.17** | **2.06** |  | 148 | **1.31** | **1.11** | **1.54** |  | 199 | **1.22** | **1.06** | **1.40** |  | 267 | **1.43** | **1.26** | **1.61** |  |
| Autoimmune hemolytic anemia | 1 | 0.92 | 0.00 | 5.26 |  | 3 | 1.05 | 0.20 | 3.09 |  | 10 | 1.34 | 0.64 | 2.47 |  | 47 | **1.42** | **1.04** | **1.89** |  |
| Behcet´s disease | 42 | **1.77** | **1.27** | **2.39** |  | 69 | 1.25 | 0.97 | 1.58 |  | 88 | 1.22 | 0.98 | 1.50 |  | 184 | **1.26** | **1.08** | **1.45** |  |
| Celiac disease | 8 | 1.21 | 0.52 | 2.39 |  | 24 | 1.29 | 0.83 | 1.92 |  | 43 | 1.08 | 0.78 | 1.45 |  | 82 | 0.86 | 0.69 | 1.07 |  |
| Chorea minor | 0 |  |  |  |  | 3 | **42.86** | **8.08** | **126.86** |  | 1 | 2.56 | 0.00 | 14.70 |  | 2 | 1.68 | 0.16 | 6.18 |  |
| Crohn´s disease | 57 | 1.07 | 0.81 | 1.38 |  | 111 | 0.75 | 0.62 | 0.91 |  | 161 | 0.84 | 0.71 | 0.98 |  | 298 | 1.02 | 0.91 | 1.14 |  |
| Diabetes mellitus type I | 229 | **3.15** | **2.76** | **3.59** |  | 22 | 1.27 | 0.80 | 1.93 |  | 1 | 1.47 | 0.00 | 8.43 |  | 0 |  |  |  |  |
| Discoid lupus erythematosus | 2 | 2.13 | 0.20 | 7.82 |  | 4 | 0.96 | 0.25 | 2.49 |  | 22 | **2.58** | **1.62** | **3.92** |  | 25 | **1.56** | **1.01** | **2.31** |  |
| Grave´s disease | 37 | 1.18 | 0.83 | 1.63 |  | 158 | **1.27** | **1.08** | **1.49** |  | 315 | 1.03 | 0.92 | 1.15 |  | 885 | **1.06** | **1.00** | **1.14** |  |
| Hashimoto´s thyroiditis | 8 | 1.45 | 0.62 | 2.87 |  | 48 | **2.24** | **1.65** | **2.97** |  | 92 | **1.70** | **1.37** | **2.09** |  | 332 | **1.60** | **1.43** | **1.78** |  |
| Immune thrombocytopenic purpura | 5 | 1.27 | 0.40 | 2.99 |  | 14 | 1.62 | 0.88 | 2.73 |  | 35 | **1.78** | **1.24** | **2.48** |  | 92 | **1.46** | **1.18** | **1.79** |  |
| Localized scleroderma | 1 | 1.79 | 0.00 | 10.24 |  | 1 | 0.64 | 0.00 | 3.65 |  | 3 | 0.80 | 0.15 | 2.37 |  | 15 | 1.01 | 0.56 | 1.66 |  |
| Lupoid hepatitis | 0 | 0.00 | 1.41 | 5.77 |  | 4 | 2.01 | 0.52 | 5.20 |  | 3 | 0.83 | 0.16 | 2.45 |  | 3 | 0.43 | 0.08 | 1.28 |  |
| Multiple sclerosis | 24 | **1.67** | **1.07** | **2.49** |  | 87 | **1.42** | **1.14** | **1.75** |  | 146 | **1.28** | **1.08** | **1.51** |  | 217 | 1.14 | 0.99 | 1.30 |  |
| Myasthenia gravis | 7 | **2.92** | **1.16** | **6.04** |  | 7 | 0.90 | 0.36 | 1.86 |  | 36 | **1.43** | **1.00** | **1.98** |  | 128 | **1.26** | **1.05** | **1.50** |  |
| Pernicious anemia | 3 | 1.05 | 0.20 | 3.11 |  | 27 | 1.29 | 0.85 | 1.89 |  | 114 | 1.08 | 0.89 | 1.30 |  | 1292 | **1.22** | **1.15** | **1.29** |  |
| Polyarteritis nodosa | 8 | **3.54** | **1.51** | **7.01** |  | 11 | 1.61 | 0.80 | 2.88 |  | 32 | **1.57** | **1.07** | **2.22** |  | 69 | 1.18 | 0.92 | 1.49 |  |
| Polymyalgia rheumatica | 51 | **2.02** | **1.50** | **2.66** |  | 173 | **1.75** | **1.50** | **2.03** |  | 295 | **1.29** | **1.15** | **1.45** |  | 1427 | **1.42** | **1.35** | **1.50** |  |
| Polymyositis/dermatomyositis | 2 | 1.44 | 0.14 | 5.29 |  | 9 | 1.83 | 0.83 | 3.49 |  | 20 | 1.50 | 0.91 | 2.32 |  | 56 | **1.70** | **1.29** | **2.21** |  |
| Primary biliary cirrhosis | 2 | 1.45 | 0.14 | 5.33 |  | 3 | 0.64 | 0.12 | 1.90 |  | 12 | 0.98 | 0.50 | 1.72 |  | 27 | **1.67** | **1.10** | **2.43** |  |
| Psoriasis | 92 | **1.80** | **1.45** | **2.21** |  | 330 | **1.84** | **1.65** | **2.05** |  | 541 | **1.58** | **1.45** | **1.72** |  | 1006 | **1.44** | **1.35** | **1.53** |  |
| Reiter´s disease | 4 | 2.33 | 0.60 | 6.01 |  | 9 | 2.01 | 0.91 | 3.83 |  | 12 | **2.83** | **1.46** | **4.96** |  | 5 | 0.68 | 0.21 | 1.60 |  |
| Rheumatic fever | 62 | **1.96** | **1.50** | **2.52** |  | 119 | **1.61** | **1.34** | **1.93** |  | 147 | **1.31** | **1.11** | **1.54** |  | 211 | **1.22** | **1.06** | **1.39** |  |
| Rheumatoid arthritis | 56 | **2.42** | **1.83** | **3.14** |  | 267 | **2.15** | **1.90** | **2.43** |  | 968 | **2.23** | **2.09** | **2.38** |  | 2817 | **1.75** | **1.68** | **1.81** |  |
| Sarcoidosis | 51 | 1.03 | 0.76 | 1.35 |  | 137 | 1.00 | 0.84 | 1.18 |  | 219 | **1.14** | **1.00** | **1.30** |  | 306 | 1.05 | 0.94 | 1.18 |  |
| Sjögren´s syndrome | 1 | 2.63 | 0.00 | 15.08 |  | 0 |  |  |  |  | 8 | 2.25 | 0.96 | 4.45 |  | 18 | **1.93** | **1.14** | **3.06** |  |
| Systemic lupus erythematosus | 17 | **3.63** | **2.11** | **5.83** |  | 31 | **2.39** | **1.63** | **3.40** |  | 55 | **1.94** | **1.46** | **2.53** |  | 132 | **1.74** | **1.46** | **2.07** |  |
| Systemic sclerosis | 18 | 0.99 | 0.58 | 1.57 |  | 65 | **1.43** | **1.10** | **1.82** |  | 125 | **1.42** | **1.19** | **1.70** |  | 315 | **1.18** | **1.06** | **1.32** |  |
| Ulcerative colitis | 88 | 1.03 | 0.83 | 1.27 |  | 246 | 1.06 | 0.93 | 1.20 |  | 338 | 1.01 | 0.91 | 1.12 |  | 845 | **1.32** | **1.23** | **1.41** |  |
| Wegener´s granulomatosis | 3 | 1.08 | 0.20 | 3.19 |  | 29 | **1.71** | **1.15** | **2.46** |  | 164 | **1.54** | **1.31** | **1.80** |  | 1232 | **1.36** | **1.28** | **1.44** |  |
| All | 939 | **1.68** | **1.58** | **1.80** |  | 2193 | **1.40** | **1.34** | **1.46** |  | 4274 | **1.39** | **1.35** | **1.43** |  | 12528 | **1.37** | **1.34** | **1.39** |  |
| O = observed number of cases; SIR = standardized incidence ratio; CI = confidence interval. | | | | | | | | | |  |  |  |  |  |  |  |  |  |  |  |
| Bold type: 95% CI does not include 1.00. |  |  |  |  |  |  |  |  |  |  |  |  |  |  |  |  |  |  |  |  |
| Adjusted for age, period, socioeconomic status, hospitalization of chronic lower respiratory diseases, obesity, alcoholism, hypertension, diabetes, arterial flutter, heart failure, and renal disease. | | | | | | | | | | | | | | | | | |  |  |  |
