# Supplementary material for: Risk of Subsequent Coronary Heart Disease in Patients Hospitalized for Immune-Mediated Diseases: A Nationwide Follow-Up Study from Sweden
Source: PLoS One. 2012 Mar 16;7(3):e33442. doi: 10.1371/journal.pone.0033442 (PMC3306397; doi:10.1371/journal.pone.0033442)
Supplement: Table S6 — SIR for subsequent CHD of female patients with IMD after one year of follow-up. (DOC) [file pone.0033442.s006.doc]

| **Table S6. SIR for subsequent CHD of female patients with IMD after one year of follow-up** | | | | | | | | | | | | | | | | | | | |  |
| --- | --- | --- | --- | --- | --- | --- | --- | --- | --- | --- | --- | --- | --- | --- | --- | --- | --- | --- | --- | --- |
|  | Age at diagnosis of CHD (years) | | | | | | | | | | | | | | | | | | |  |
|  | <50 | | | |  | 50-59 | | | |  | 60-69 | | | |  | >=70 | | | |  |
| Immune-mediated diseases | O | SIR | 95% CI | |  | O | SIR | 95% CI | |  | O | SIR | 95% CI | |  | O | SIR | 95% CI | |  |
| Addison´s disease | 3 | 1.68 | 0.32 | 4.96 |  | 7 | 1.43 | 0.57 | 2.97 |  | 17 | 1.67 | 0.97 | 2.68 |  | 70 | **1.35** | **1.05** | **1.70** |  |
| Amyotrophic lateral sclerosis | 0 |  |  |  |  | 7 | **4.32** | **1.71** | **8.95** |  | 9 | **1.51** | **0.68** | **2.87** |  | 88 | **1.92** | **1.54** | **2.36** |  |
| Ankylosing spondylitis | 7 | 1.73 | 0.69 | 3.59 |  | 25 | 1.50 | 0.97 | 2.21 |  | 29 | **1.03** | **0.69** | **1.48** |  | 73 | 1.13 | 0.89 | 1.42 |  |
| Autoimmune hemolytic anemia | 0 |  |  |  |  | 0 |  |  |  |  | 8 | 1.61 | 0.69 | 3.19 |  | 77 | **1.61** | **1.27** | **2.01** |  |
| Behcet´s disease | 6 | 2.29 | 0.82 | 5.02 |  | 12 | 1.66 | 0.85 | 2.91 |  | 26 | **1.62** | **1.06** | **2.37** |  | 184 | **1.56** | **1.34** | **1.80** |  |
| Celiac disease | 4 | 0.88 | 0.23 | 2.26 |  | 12 | 1.02 | 0.52 | 1.78 |  | 32 | 1.18 | 0.81 | 1.67 |  | 144 | 1.25 | 1.06 | 1.48 |  |
| Chorea minor | 1 | 25.00 | 0.01 | 143.31 |  | 0 |  |  |  |  | 1 | 5.88 | 0.00 | 33.72 |  | 10 | **3.37** | **1.60** | **6.22** |  |
| Crohn´s disease | 13 | 0.65 | 0.35 | 1.12 |  | 62 | 1.11 | 0.85 | 1.43 |  | 104 | 1.12 | 0.92 | 1.36 |  | 363 | **1.16** | **1.04** | **1.29** |  |
| Diabetes mellitus type I | 224 | **3.92** | **3.43** | **4.47** |  | 36 | **1.99** | **1.39** | **2.75** |  | 0 |  |  |  |  | 0 |  |  |  |  |
| Discoid lupus erythematosus | 1 | 1.23 | 0.00 | 7.08 |  | 12 | **3.51** | **1.80** | **6.15** |  | 19 | **2.68** | **1.61** | **4.19** |  | 36 | **1.49** | **1.04** | **2.06** |  |
| Grave´s disease | 78 | **1.32** | **1.04** | **1.64** |  | 267 | **1.21** | **1.07** | **1.36** |  | 769 | **1.26** | **1.17** | **1.35** |  | 4240 | **1.21** | **1.17** | **1.24** |  |
| Hashimoto´s thyroiditis | 13 | 1.63 | 0.86 | 2.79 |  | 85 | **2.38** | **1.90** | **2.95** |  | 243 | **1.98** | **1.74** | **2.25** |  | 1389 | **1.57** | **1.49** | **1.66** |  |
| Immune thrombocytopenic purpura | 4 | 2.03 | 0.53 | 5.25 |  | 5 | 1.02 | 0.32 | 2.39 |  | 22 | **1.96** | **1.23** | **2.98** |  | 118 | **1.29** | **1.06** | **1.54** |  |
| Localized scleroderma | 0 |  |  |  |  | 3 | 1.51 | 0.28 | 4.46 |  | 7 | 0.66 | 0.26 | 1.36 |  | 151 | **1.31** | **1.11** | **1.54** |  |
| Lupoid hepatitis | 1 | 2.22 | 0.00 | 12.74 |  | 2 | 1.74 | 0.16 | 6.40 |  | 5 | 2.33 | 0.73 | 5.47 |  | 11 | 1.00 | 0.50 | 1.80 |  |
| Multiple sclerosis | 23 | **2.34** | **1.48** | **3.52** |  | 64 | **1.53** | **1.18** | **1.95** |  | 130 | **1.55** | **1.30** | **1.84** |  | 278 | 1.02 | 0.91 | 1.15 |  |
| Myasthenia gravis | 3 | 1.38 | 0.26 | 4.09 |  | 10 | 1.70 | 0.81 | 3.13 |  | 16 | 1.32 | 0.76 | 2.16 |  | 109 | **1.31** | **1.07** | **1.58** |  |
| Pernicious anemia | 0 |  |  |  |  | 12 | 1.59 | 0.82 | 2.79 |  | 62 | **1.31** | **1.01** | **1.69** |  | 1712 | **1.46** | **1.39** | **1.53** |  |
| Polyarteritis nodosa | 1 | 1.92 | 0.00 | 11.02 |  | 7 | **2.57** | **1.02** | **5.33** |  | 11 | 1.65 | 0.82 | 2.96 |  | 69 | **1.61** | **1.25** | **2.04** |  |
| Polymyalgia rheumatica | 27 | **2.54** | **1.67** | **3.69** |  | 135 | **2.49** | **2.09** | **2.94** |  | 287 | **1.67** | **1.48** | **1.87** |  | 2806 | **1.57** | **1.51** | **1.62** |  |
| Polymyositis/dermatomyositis | 2 | 2.60 | 0.24 | 9.55 |  | 11 | **3.55** | **1.76** | **6.37** |  | 16 | **1.80** | **1.02** | **2.93** |  | 68 | **1.73** | **1.34** | **2.19** |  |
| Primary biliary cirrhosis | 3 | 4.48 | 0.84 | 13.25 |  | 6 | 1.90 | 0.69 | 4.17 |  | 15 | 1.39 | 0.77 | 2.29 |  | 56 | **1.67** | **1.26** | **2.16** |  |
| Psoriasis | 29 | 1.30 | 0.87 | 1.86 |  | 127 | **2.20** | **1.83** | **2.61** |  | 242 | **1.82** | **1.60** | **2.06** |  | 1053 | **1.53** | **1.44** | **1.63** |  |
| Reiter´s disease | 0 |  |  |  |  | 0 | 0.00 | 6.40 | 26.14 |  | 0 |  |  |  |  | 2 | 0.71 | 0.07 | 2.63 |  |
| Rheumatic fever | 7 | **3.17** | **1.26** | **6.56** |  | 20 | **2.42** | **1.48** | **3.75** |  | 37 | **1.77** | **1.25** | **2.45** |  | 136 | **1.44** | **1.21** | **1.71** |  |
| Rheumatoid arthritis | 54 | **2.91** | **2.19** | **3.80** |  | 238 | **2.55** | **2.24** | **2.89** |  | 1075 | **2.74** | **2.57** | **2.90** |  | 6426 | **1.85** | **1.80** | **1.89** |  |
| Sarcoidosis | 16 | 1.52 | 0.86 | 2.47 |  | 50 | 1.18 | 0.88 | 1.56 |  | 147 | **1.20** | **1.02** | **1.41** |  | 555 | 1.07 | 0.98 | 1.16 |  |
| Sjögren´s syndrome | 2 | 2.20 | 0.21 | 8.08 |  | 9 | 1.58 | 0.72 | 3.02 |  | 43 | **2.31** | **1.67** | **3.12** |  | 118 | **1.39** | **1.15** | **1.67** |  |
| Systemic lupus erythematosus | 49 | **7.53** | **5.57** | **9.96** |  | 83 | **3.38** | **2.69** | **4.19** |  | 145 | **2.63** | **2.22** | **3.10** |  | 358 | **1.73** | **1.55** | **1.91** |  |
| Systemic sclerosis | 10 | **3.85** | **1.83** | **7.10** |  | 11 | 1.30 | 0.65 | 2.33 |  | 62 | **2.03** | **1.55** | **2.60** |  | 337 | **1.43** | **1.28** | **1.59** |  |
| Ulcerative colitis | 34 | 1.41 | 0.97 | 1.97 |  | 73 | 1.09 | 0.85 | 1.37 |  | 136 | **1.21** | **1.01** | **1.43** |  | 603 | **1.15** | **1.06** | **1.25** |  |
| Wegener´s granulomatosis | 2 | 2.38 | 0.22 | 8.76 |  | 19 | **3.11** | **1.87** | **4.87** |  | 115 | **1.39** | **1.15** | **1.67** |  | 2820 | **1.51** | **1.46** | **1.57** |  |
| All | 617 | **2.23** | **2.06** | **2.42** |  | 1410 | **1.72** | **1.64** | **1.82** |  | 3830 | **1.69** | **1.64** | **1.75** |  | 24460 | **1.48** | **1.46** | **1.50** |  |
| O = observed number of cases; SIR = standardized incidence ratio; CI = confidence interval. | | | | | | | | | |  |  |  |  |  |  |  |  |  |  |  |
| Bold type: 95% CI does not include 1.00. |  |  |  |  |  |  |  |  |  |  |  |  |  |  |  |  |  |  |  |  |
| Adjusted for age, period, socioeconomic status, hospitalization of chronic lower respiratory diseases, obesity, alcoholism, hypertension, diabetes, arterial flutter, heart failure, and renal disease. | | | | | | | | | | | | | | | | | | | |  |
