# Supplementary material for: Risk of Subsequent Coronary Heart Disease in Patients Hospitalized for Immune-Mediated Diseases: A Nationwide Follow-Up Study from Sweden
Source: PLoS One. 2012 Mar 16;7(3):e33442. doi: 10.1371/journal.pone.0033442 (PMC3306397; doi:10.1371/journal.pone.0033442)
Supplement: Table S7 — SIR for subsequent CHD of patients with IMD after one year of follow-up for time periods 1964–1993 and 1994–2008. (DOC) [file pone.0033442.s007.doc]

| **Table S7. SIR for subsequent CHD of patients with IMD after one year of follow-up** | | | | | | | | | | | |
| --- | --- | --- | --- | --- | --- | --- | --- | --- | --- | --- | --- |
|  | Period of diagnosis (years) | | | | | | | | |  |  |
|  | 1964-1993 | | | |  | 1994-2008 | | | |  |  |
| Immune-mediated diseases | O | SIR | 95% CI | |  | O | SIR | 95% CI | |  |  |
| Addison´s disease | 39 | **2.27** | **1.61** | **3.11** |  | 164 | **1.37** | **1.17** | **1.60** |  |  |
| Amyotrophic lateral sclerosis | 228 | **1.85** | **1.62** | **2.11** |  | 73 | **1.39** | **1.09** | **1.75** |  |  |
| Ankylosing spondylitis | 281 | **1.40** | **1.24** | **1.57** |  | 384 | **1.22** | **1.10** | **1.35** |  |  |
| Autoimmune hemolytic anemia | 65 | **1.46** | **1.13** | **1.86** |  | 83 | **1.57** | **1.25** | **1.94** |  |  |
| Behcet´s disease | 379 | **1.58** | **1.43** | **1.75** |  | 80 | **1.29** | **1.02** | **1.61** |  |  |
| Celiac disease | 126 | 1.11 | 0.93 | 1.32 |  | 5602 | **1.21** | **1.18** | **1.25** |  |  |
| Chorea minor | 15 | **3.64** | **2.03** | **6.02** |  | 226 | 0.82 | 0.72 | 0.93 |  |  |
| Crohn´s disease | 294 | 1.08 | 0.96 | 1.21 |  | 1210 | **1.16** | **1.10** | **1.23** |  |  |
| Diabetes mellitus type I | 98 | **3.41** | **2.76** | **4.15** |  | 213 | **3.07** | **2.67** | **3.51** |  |  |
| Discoid lupus erythematosus | 69 | **2.09** | **1.62** | **2.64** |  | 448 | 0.99 | 0.90 | 1.09 |  |  |
| Grave´s disease | 3519 | **1.32** | **1.27** | **1.36** |  | 2918 | **1.09** | **1.05** | **1.13** |  |  |
| Hashimoto´s thyroiditis | 1213 | **1.82** | **1.72** | **1.92** |  | 915 | **1.49** | **1.39** | **1.59** |  |  |
| Immune thrombocytopenic purpura | 114 | **1.58** | **1.31** | **1.90** |  | 371 | **1.92** | **1.73** | **2.13** |  |  |
| Localized scleroderma | 65 | 1.10 | 0.85 | 1.40 |  | 113 | **1.32** | **1.08** | **1.58** |  |  |
| Lupoid hepatitis | 16 | 1.00 | 0.57 | 1.63 |  | 140 | 1.15 | 0.97 | 1.36 |  |  |
| Multiple sclerosis | 396 | **1.38** | **1.25** | **1.52** |  | 485 | **1.15** | **1.05** | **1.26** |  |  |
| Myasthenia gravis | 118 | **1.54** | **1.28** | **1.85** |  | 401 | **1.28** | **1.16** | **1.41** |  |  |
| Pernicious anemia | 2371 | **1.33** | **1.28** | **1.39** |  | 640 | **1.31** | **1.21** | **1.42** |  |  |
| Polyarteritis nodosa | 88 | **1.69** | **1.36** | **2.08** |  | 94 | **1.38** | **1.11** | **1.69** |  |  |
| Polymyalgia rheumatica | 1563 | **1.54** | **1.46** | **1.61** |  | 3692 | **1.55** | **1.50** | **1.60** |  |  |
| Polymyositis/dermatomyositis | 86 | **1.87** | **1.49** | **2.31** |  | 713 | **1.56** | **1.44** | **1.67** |  |  |
| Primary biliary cirrhosis | 73 | **1.66** | **1.30** | **2.08** |  | 345 | **1.36** | **1.22** | **1.51** |  |  |
| Psoriasis | 1701 | **1.73** | **1.65** | **1.81** |  | 1120 | **1.49** | **1.40** | **1.58** |  |  |
| Reiter´s disease | 6 | 2.14 | 0.77 | 4.68 |  | 252 | **1.20** | **1.06** | **1.36** |  |  |
| Rheumatic fever | 395 | **1.64** | **1.48** | **1.81** |  | 417 | 1.06 | 0.96 | 1.16 |  |  |
| Rheumatoid arthritis | 6094 | **2.16** | **2.11** | **2.22** |  | 5178 | **1.76** | **1.71** | **1.81** |  |  |
| Sarcoidosis | 653 | **1.20** | **1.11** | **1.29** |  | 1045 | 1.04 | 0.98 | 1.11 |  |  |
| Sjögren´s syndrome | 61 | **2.11** | **1.61** | **2.71** |  | 181 | **1.44** | **1.24** | **1.67** |  |  |
| Systemic lupus erythematosus | 424 | **2.47** | **2.24** | **2.72** |  | 565 | **1.55** | **1.42** | **1.68** |  |  |
| Systemic sclerosis | 572 | **1.41** | **1.30** | **1.53** |  | 215 | **1.43** | **1.25** | **1.64** |  |  |
| Ulcerative colitis | 739 | **1.29** | **1.20** | **1.38** |  | 1148 | **1.14** | **1.08** | **1.21** |  |  |
| Wegener´s granulomatosis | 3059 | **1.43** | **1.38** | **1.48** |  | 1311 | **1.54** | **1.45** | **1.62** |  |  |
| All | 24920 | **1.58** | **1.56** | **1.60** |  | 19899 | **1.42** | **1.40** | **1.44** |  |  |
| O = observed number of cases; SIR = standardized incidence ratio; CI = confidence interval. | | | | | | | | | |  |  |
| Bold type: 95% CI does not include 1.00. |  |  |  |  |  |  |  |  |  |  |  |
| Adjusted for age, period, socioeconomic status, hospitalization of chronic lower respiratory diseases, obesity, alcoholism, hypertension, diabetes, arterial flutter, heart failure, and renal disease. | | | | | | | | | | | |
