# Supplementary material for: Risk of Subsequent Coronary Heart Disease in Patients Hospitalized for Immune-Mediated Diseases: A Nationwide Follow-Up Study from Sweden
Source: PLoS One. 2012 Mar 16;7(3):e33442. doi: 10.1371/journal.pone.0033442 (PMC3306397; doi:10.1371/journal.pone.0033442)
Supplement: Table S8 — SIR for subsequent CHD of male patients with IMD after one year of follow-up for time periods 1964–1993 and 1994–2008. (DOC) [file pone.0033442.s008.doc]

| **Table S8. SIR for subsequent CHD of male patients with IMD after one year of follow-up** | | | | | | | | | | | |  |
| --- | --- | --- | --- | --- | --- | --- | --- | --- | --- | --- | --- | --- |
|  | Period of diagnosis (years) | | | | | | | | |  |  | |
|  | 1964-1993 | | | |  | 1994-2008 | | | |  |  | |
| Immune-mediated diseases | O | SIR | 95% CI | |  | O | SIR | 95% CI | |  |  | |
| Addison´s disease | 20 | **2.57** | **1.57** | **3.97** |  | 86 | **1.43** | **1.15** | **1.77** |  |  | |
| Amyotrophic lateral sclerosis | 156 | **1.73** | **1.47** | **2.03** |  | 41 | 1.30 | 0.93 | 1.77 |  |  | |
| Ankylosing spondylitis | 236 | **1.35** | **1.18** | **1.53** |  | 295 | **1.30** | **1.15** | **1.45** |  |  | |
| Autoimmune hemolytic anemia | 23 | 1.32 | 0.83 | 1.98 |  | 40 | **1.59** | **1.14** | **2.17** |  |  | |
| Behcet´s disease | 220 | **1.46** | **1.27** | **1.67** |  | 11 | 1.61 | 0.80 | 2.88 |  |  | |
| Celiac disease | 56 | 0.94 | 0.71 | 1.22 |  | 5480 | **1.21** | **1.18** | **1.25** |  |  | |
| Chorea minor | 6 | **3.92** | **1.41** | **8.59** |  | 223 | 0.81 | 0.71 | 0.92 |  |  | |
| Crohn´s disease | 177 | 1.08 | 0.93 | 1.25 |  | 785 | **1.17** | **1.09** | **1.26** |  |  | |
| Diabetes mellitus type I | 45 | **3.29** | **2.40** | **4.41** |  | 6 | 0.67 | 0.24 | 1.47 |  |  | |
| Discoid lupus erythematosus | 33 | **1.79** | **1.23** | **2.52** |  | 416 | 0.97 | 0.88 | 1.07 |  |  | |
| Grave´s disease | 754 | **1.18** | **1.09** | **1.26** |  | 329 | 1.11 | 0.99 | 1.24 |  |  | |
| Hashimoto´s thyroiditis | 274 | **1.81** | **1.60** | **2.03** |  | 124 | **1.52** | **1.26** | **1.81** |  |  | |
| Immune thrombocytopenic purpura | 50 | **1.53** | **1.14** | **2.02** |  | 286 | **2.33** | **2.07** | **2.62** |  |  | |
| Localized scleroderma | 13 | 1.53 | 0.81 | 2.62 |  | 4 | 0.48 | 0.12 | 1.23 |  |  | |
| Lupoid hepatitis | 7 | 0.89 | 0.35 | 1.84 |  | 130 | 1.13 | 0.94 | 1.34 |  |  | |
| Multiple sclerosis | 192 | **1.27** | **1.09** | **1.46** |  | 194 | **1.30** | **1.12** | **1.49** |  |  | |
| Myasthenia gravis | 63 | **1.44** | **1.11** | **1.85** |  | 318 | **1.31** | **1.17** | **1.46** |  |  | |
| Pernicious anemia | 1035 | **1.20** | **1.13** | **1.28** |  | 190 | 1.10 | 0.95 | 1.27 |  |  | |
| Polyarteritis nodosa | 52 | **1.48** | **1.10** | **1.94** |  | 42 | 1.30 | 0.94 | 1.76 |  |  | |
| Polymyalgia rheumatica | 673 | **1.45** | **1.34** | **1.56** |  | 1327 | **1.46** | **1.38** | **1.54** |  |  | |
| Polymyositis/dermatomyositis | 48 | **1.94** | **1.43** | **2.58** |  | 654 | **1.53** | **1.41** | **1.65** |  |  | |
| Primary biliary cirrhosis | 30 | 1.44 | 0.97 | 2.05 |  | 308 | **1.35** | **1.20** | **1.50** |  |  | |
| Psoriasis | 1006 | **1.66** | **1.56** | **1.77** |  | 364 | **1.56** | **1.41** | **1.73** |  |  | |
| Reiter´s disease | 5 | 1.92 | 0.61 | 4.52 |  | 251 | **1.22** | **1.07** | **1.38** |  |  | |
| Rheumatic fever | 283 | **1.57** | **1.39** | **1.76** |  | 329 | 1.00 | 0.89 | 1.11 |  |  | |
| Rheumatoid arthritis | 2242 | **2.06** | **1.97** | **2.15** |  | 1237 | **1.79** | **1.69** | **1.89** |  |  | |
| Sarcoidosis | 292 | **1.13** | **1.01** | **1.27** |  | 638 | 1.07 | 0.99 | 1.15 |  |  | |
| Sjögren´s syndrome | 8 | 1.48 | 0.63 | 2.93 |  | 62 | **1.58** | **1.21** | **2.02** |  |  | |
| Systemic lupus erythematosus | 118 | **2.08** | **1.72** | **2.49** |  | 236 | **1.27** | **1.11** | **1.44** |  |  | |
| Systemic sclerosis | 322 | **1.32** | **1.18** | **1.47** |  | 45 | 1.36 | 0.99 | 1.83 |  |  | |
| Ulcerative colitis | 487 | **1.30** | **1.19** | **1.42** |  | 554 | **1.16** | **1.06** | **1.26** |  |  | |
| Wegener´s granulomatosis | 1055 | **1.41** | **1.32** | **1.50** |  | 359 | **1.22** | **1.10** | **1.36** |  |  | |
| All | 9981 | **1.49** | **1.46** | **1.52** |  | 4521 | **1.42** | **1.38** | **1.46** |  |  | |
| O = observed number of cases; SIR = standardized incidence ratio; CI = confidence interval. | | | | | | | | | |  |  | |
| Bold type: 95% CI does not include 1.00. |  |  |  |  |  |  |  |  |  |  |  | |
| Adjusted for age, period, socioeconomic status, hospitalization of chronic lower respiratory diseases, obesity, alcoholism, hypertension, diabetes, arterial flutter, heart failure, and renal disease. | | | | | | | | | | | |  |
