# Supplementary material for: Risk of Subsequent Coronary Heart Disease in Patients Hospitalized for Immune-Mediated Diseases: A Nationwide Follow-Up Study from Sweden
Source: PLoS One. 2012 Mar 16;7(3):e33442. doi: 10.1371/journal.pone.0033442 (PMC3306397; doi:10.1371/journal.pone.0033442)
Supplement: Table S9 — SIR for subsequent CHD of female patients with IMD after one year of follow-up for time periods 1964–1993 and 1994–2008. (DOC) [file pone.0033442.s009.doc]

| **Table S9. SIR for subsequent CHD of female patients with IMD after one year of follow-up** | | | | | | | | | |  |
| --- | --- | --- | --- | --- | --- | --- | --- | --- | --- | --- |
|  | Period of diagnosis (years) | | | | | | | | |  |
|  | 1964-1993 | | | |  | 1994-2008 | | | |  |
| Immune-mediated diseases | O | SIR | 95% CI | |  | O | SIR | 95% CI | |  |
| Addison´s disease | 19 | **2.03** | **1.22** | **3.17** |  | 78 | **1.31** | **1.04** | **1.64** |  |
| Amyotrophic lateral sclerosis | 72 | **2.19** | **1.71** | **2.75** |  | 32 | **1.53** | **1.05** | **2.16** |  |
| Ankylosing spondylitis | 45 | **1.70** | **1.24** | **2.28** |  | 89 | 1.02 | 0.82 | 1.26 |  |
| Autoimmune hemolytic anemia | 42 | **1.56** | **1.12** | **2.11** |  | 43 | **1.55** | **1.12** | **2.08** |  |
| Behcet´s disease | 159 | **1.79** | **1.52** | **2.09** |  | 69 | 1.25 | 0.97 | 1.58 |  |
| Celiac disease | 70 | **1.30** | **1.01** | **1.64** |  | 122 | 1.17 | 0.97 | 1.40 |  |
| Chorea minor | 9 | **3.47** | **1.58** | **6.62** |  | 3 | 4.48 | 0.84 | 13.25 |  |
| Crohn´s disease | 117 | 1.08 | 0.90 | 1.30 |  | 425 | **1.14** | **1.03** | **1.25** |  |
| Diabetes mellitus type I | 53 | **3.51** | **2.63** | **4.59** |  | 207 | **3.42** | **2.97** | **3.92** |  |
| Discoid lupus erythematosus | 36 | **2.46** | **1.72** | **3.41** |  | 32 | **1.53** | **1.05** | **2.16** |  |
| Grave´s disease | 2765 | **1.36** | **1.31** | **1.41** |  | 2589 | **1.09** | **1.05** | **1.13** |  |
| Hashimoto´s thyroiditis | 939 | **1.82** | **1.71** | **1.94** |  | 791 | **1.48** | **1.38** | **1.59** |  |
| Immune thrombocytopenic purpura | 64 | **1.63** | **1.25** | **2.08** |  | 85 | 1.21 | 0.96 | 1.49 |  |
| Localized scleroderma | 52 | 1.03 | 0.77 | 1.35 |  | 109 | **1.41** | **1.16** | **1.70** |  |
| Lupoid hepatitis | 9 | 1.12 | 0.51 | 2.13 |  | 10 | 1.50 | 0.71 | 2.77 |  |
| Multiple sclerosis | 204 | **1.50** | **1.31** | **1.73** |  | 291 | 1.07 | 0.95 | 1.20 |  |
| Myasthenia gravis | 55 | **1.67** | **1.26** | **2.18** |  | 83 | 1.18 | 0.94 | 1.46 |  |
| Pernicious anemia | 1336 | **1.46** | **1.38** | **1.54** |  | 450 | **1.42** | **1.29** | **1.56** |  |
| Polyarteritis nodosa | 36 | **2.13** | **1.49** | **2.95** |  | 52 | **1.45** | **1.08** | **1.90** |  |
| Polymyalgia rheumatica | 890 | **1.61** | **1.51** | **1.72** |  | 2365 | **1.60** | **1.54** | **1.67** |  |
| Polymyositis/dermatomyositis | 38 | **1.78** | **1.26** | **2.45** |  | 59 | **1.92** | **1.46** | **2.48** |  |
| Primary biliary cirrhosis | 43 | **1.85** | **1.34** | **2.50** |  | 37 | **1.48** | **1.04** | **2.04** |  |
| Psoriasis | 695 | **1.83** | **1.70** | **1.98** |  | 756 | **1.45** | **1.35** | **1.56** |  |
| Reiter´s disease | 1 | 4.76 | 0.00 | 27.30 |  | 1 | 0.28 | 0.00 | 1.63 |  |
| Rheumatic fever | 112 | **1.84** | **1.52** | **2.22** |  | 88 | **1.36** | **1.09** | **1.68** |  |
| Rheumatoid arthritis | 3852 | **2.22** | **2.16** | **2.30** |  | 3941 | **1.75** | **1.70** | **1.81** |  |
| Sarcoidosis | 361 | **1.25** | **1.13** | **1.39** |  | 407 | 1.01 | 0.91 | 1.11 |  |
| Sjögren´s syndrome | 53 | **2.25** | **1.69** | **2.95** |  | 119 | **1.38** | **1.14** | **1.65** |  |
| Systemic lupus erythematosus | 306 | **2.66** | **2.37** | **2.98** |  | 329 | **1.84** | **1.65** | **2.05** |  |
| Systemic sclerosis | 250 | **1.56** | **1.37** | **1.76** |  | 170 | **1.45** | **1.24** | **1.68** |  |
| Ulcerative colitis | 252 | **1.26** | **1.11** | **1.42** |  | 594 | **1.13** | **1.04** | **1.22** |  |
| Wegener´s granulomatosis | 2004 | **1.44** | **1.37** | **1.50** |  | 952 | **1.70** | **1.59** | **1.81** |  |
| All | 14939 | **1.65** | **1.62** | **1.67** |  | 15378 | **1.42** | **1.40** | **1.44** |  |
| O = observed number of cases; SIR = standardized incidence ratio; CI = confidence interval. | | | | | | | | | |  |
| Bold type: 95% CI does not include 1.00. |  |  |  |  |  |  |  |  |  |  |
| Adjusted for age, period, socioeconomic status, hospitalization of chronic lower respiratory diseases, obesity, alcoholism, hypertension, diabetes, arterial flutter, heart failure, and renal disease. | | | | | | | | | | |
