# Supplementary material for: Risk of Subsequent Coronary Heart Disease in Patients Hospitalized for Immune-Mediated Diseases: A Nationwide Follow-Up Study from Sweden
Source: PLoS One. 2012 Mar 16;7(3):e33442. doi: 10.1371/journal.pone.0033442 (PMC3306397; doi:10.1371/journal.pone.0033442)
Supplement: Table S10 — SIR for all subsequent CHD of patients with IMD hospitalization length <7 days or 7 or more days. (DOC) [file pone.0033442.s010.doc]

| **Table S10. SIR for all subsequent CHD of patients with IMD hospitalization length <7 days or 7 or more days** | | | | | | | | | | | |
| --- | --- | --- | --- | --- | --- | --- | --- | --- | --- | --- | --- |
|  | < 7 days | | | |  | >=7 days | | | | |  |
| Immune-mediated diseases | O | SIR | 95% CI | |  | O |  | SIR | 95% CI | |  |
| Addison´s disease | 127 | **1,50** | **1,25** | **1,78** |  | 85 |  | **1,75** | **1,40** | **2,16** |  |
| Amyotrophic lateral sclerosis | 150 | **1,60** | **1,35** | **1,88** |  | 332 |  | **2,05** | **1,83** | **2,28** |  |
| Ankylosing spondylitis | 231 | **1,59** | **1,39** | **1,81** |  | 622 |  | **1,28** | **1,18** | **1,39** |  |
| Autoimmune hemolytic anemia | 44 | **1,60** | **1,16** | **2,15** |  | 134 |  | **1,63** | **1,37** | **1,93** |  |
| Behcet´s disease | 280 | **1,30** | **1,15** | **1,46** |  | 386 |  | **1,58** | **1,43** | **1,75** |  |
| Celiac disease | 173 | 1,10 | 0,94 | 1,28 |  | 213 |  | **1,21** | **1,06** | **1,39** |  |
| Chorea minor | 3 | **6,38** | **1,20** | **18,89** |  | 18 |  | **3,64** | **2,15** | **5,77** |  |
| Crohn´s disease | 575 | **1,12** | **1,03** | **1,21** |  | 729 |  | 1,02 | 0,95 | 1,10 |  |
| Diabetes mellitus type I | 238 | **2,80** | **2,46** | **3,18** |  | 274 |  | **3,37** | **2,98** | **3,79** |  |
| Discoid lupus erythematosus | 34 | **1,88** | **1,30** | **2,63** |  | 92 |  | **1,85** | **1,49** | **2,27** |  |
| Grave´s disease | 3748 | **1,17** | **1,13** | **1,21** |  | 3475 |  | **1,29** | **1,25** | **1,33** |  |
| Hashimoto´s thyroiditis | 909 | **1,59** | **1,49** | **1,70** |  | 1676 |  | **1,96** | **1,87** | **2,06** |  |
| Immune thrombocytopenic purpura | 147 | **1,52** | **1,29** | **1,79** |  | 194 |  | **1,54** | **1,33** | **1,78** |  |
| Localized scleroderma | 111 | 1,16 | 0,96 | 1,40 |  | 81 |  | **1,36** | **1,08** | **1,69** |  |
| Lupoid hepatitis | 6 | 0,59 | 0,21 | 1,30 |  | 27 |  | 1,38 | 0,91 | 2,01 |  |
| Multiple sclerosis | 335 | **1,30** | **1,17** | **1,45** |  | 784 |  | **1,35** | **1,26** | **1,45** |  |
| Myasthenia gravis | 174 | **1,30** | **1,12** | **1,51** |  | 189 |  | **1,50** | **1,30** | **1,74** |  |
| Pernicious anemia | 656 | **1,24** | **1,14** | **1,34** |  | 2860 |  | **1,40** | **1,35** | **1,45** |  |
| Polyarteritis nodosa | 93 | **1,68** | **1,35** | **2,06** |  | 155 |  | **1,62** | **1,37** | **1,89** |  |
| Polymyalgia rheumatica | 2734 | **1,52** | **1,46** | **1,58** |  | 2935 |  | **1,63** | **1,57** | **1,69** |  |
| Polymyositis/dermatomyositis | 55 | **1,81** | **1,36** | **2,35** |  | 162 |  | **1,96** | **1,67** | **2,28** |  |
| Primary biliary cirrhosis | 65 | **1,60** | **1,24** | **2,05** |  | 96 |  | **1,80** | **1,46** | **2,20** |  |
| Psoriasis | 629 | **1,56** | **1,44** | **1,69** |  | 3096 |  | **1,66** | **1,60** | **1,71** |  |
| Reiter´s disease | 16 | 1,34 | 0,76 | 2,18 |  | 18 |  | **1,70** | **1,01** | **2,69** |  |
| Rheumatic fever | 400 | **1,46** | **1,32** | **1,61** |  | 397 |  | **1,57** | **1,42** | **1,73** |  |
| Rheumatoid arthritis | 3491 | **1,83** | **1,77** | **1,89** |  | 10498 |  | **2,18** | **2,13** | **2,22** |  |
| Sarcoidosis | 763 | **1,13** | **1,05** | **1,21** |  | 842 |  | **1,16** | **1,08** | **1,24** |  |
| Sjögren´s syndrome | 101 | **1,51** | **1,23** | **1,83** |  | 116 |  | **1,76** | **1,46** | **2,12** |  |
| Systemic lupus erythematosus | 372 | **2,35** | **2,12** | **2,60** |  | 636 |  | **2,23** | **2,06** | **2,41** |  |
| Systemic sclerosis | 337 | **1,43** | **1,28** | **1,59** |  | 731 |  | **1,47** | **1,37** | **1,59** |  |
| Ulcerative colitis | 1274 | **1,24** | **1,17** | **1,31** |  | 1294 |  | **1,19** | **1,12** | **1,25** |  |
| Wegener´s granulomatosis | 1086 | **1,43** | **1,35** | **1,52** |  | 3631 |  | **1,53** | **1,48** | **1,58** |  |
| All | 19357 | **1,41** | **1,40** | **1,44** |  | 36778 |  | **1,63** | **1,61** | **1,64** |  |
| O = observed number of cases; SIR = standardized incidence ratio; CI = confidence interval. | | | | | | | | | | |  |
| Bold type: 95% CI does not include 1.00. |  |  |  |  |  |  |  |  |  |  |  |
| Adjusted for age, period, socioeconomic status, region of residence, hospitalization of chronic lower respiratory diseases, obesity, alcoholism, hypertension, diabetes, arterial flutter, heart failure, and renal disease. | | | | | | | | | | | |
